# Hemoglobin: A Nitric-Oxide Dioxygenase

**DOI:** 10.6064/2012/683729

**Published:** 2012-12-19

**Authors:** Paul R. Gardner

**Affiliations:** Miami Valley Biotech, 1001 E. 2nd Street, Suite 2445, Dayton, OH 45402, USA

## Abstract

Members of the hemoglobin superfamily efficiently catalyze nitric-oxide dioxygenation, and when paired with native electron donors, function as NO dioxygenases (NODs). Indeed, the NOD function has emerged as a more common and ancient function than the well-known role in O_2_ transport-storage. Novel hemoglobins possessing a NOD function continue to be discovered in diverse life forms. Unique hemoglobin structures evolved, in part, for catalysis with different electron donors. The mechanism of NOD catalysis by representative single domain hemoglobins and multidomain flavohemoglobin occurs through a multistep mechanism involving O_2_ migration to the heme pocket, O_2_ binding-reduction, NO migration, radical-radical coupling, O-atom rearrangement, nitrate release, and heme iron re-reduction. Unraveling the physiological functions of multiple NODs with varying expression in organisms and the complexity of NO as both a poison and signaling molecule remain grand challenges for the NO field. NOD knockout organisms and cells expressing recombinant NODs are helping to advance our understanding of NO actions in microbial infection, plant senescence, cancer, mitochondrial function, iron metabolism, and tissue O_2_ homeostasis. NOD inhibitors are being pursued for therapeutic applications as antibiotics and antitumor agents. Transgenic NOD-expressing plants, fish, algae, and microbes are being developed for agriculture, aquaculture, and industry.

## 1. Background and Introduction

 Nitric-oxide dioxygenases (NODs) are enzymes that efficiently convert NO and O_2_ to nitrate (1). Most, if not all, NODs are hemoglobins (Hbs), and most, if not all, Hbs have the capacity to function as NODs. Hb-NODs appear to be widely distributed in nature. In fact, the NOD function appears more common and ancient than the classic O_2_ transport-storage function, or any other function, within the Hb superfamily [1]. Nevertheless, textbook familiarity with the O_2_ transport-storage function continues to blind investigators to the enzymatic functions of various members of the Hb superfamily. For example, the genome of the nematode *Caenorhabditis elegans* encodes >33 candidate Hbs [2, 3], many of which are thought to store or transport O_2_. Multiple globins are also normally expressed in non-erythroid vertebrate cells and tissues [4, 5], and globin expression is deranged in cancer cells [6–9]. When asking the question “what are all these oxygen-binding heme proteins doing?” [10], investigators are now obliged to thoroughly consider enzyme functions, and in particular a NOD function

(1) NO+O2+e−→NO3−

NODs are one of the most recent additions to the growing family of free radical and peroxide scavenging enzymes that includes the family of peroxidases [11] and peroxiredoxins (alkyl hydroperoxide reductases) [12], catalases [13], superoxide dismutases (SODs) [14, 15], superoxide reductases [16], and NO reductases (NORs) [17]. There is also preliminary evidence for a NO dismutase in certain methanotrophs [18].

 Seminal concepts within the family of peroxide and free radical scavenging enzymes can be traced to Oskar Loew's pioneering description of “catalase”, a heme-containing enzyme with the “power of catalyzing hydrogen peroxide” [13, 19]. In 1900, Loew wrote “There seems to exist no plant and no animal which is without that enzyme [catalase]”. He also presciently wrote “One of the functions of this enzyme appears to be to prevent any accumulation of hydrogen peroxide which might be formed as a by-product in the series of energetic oxidations that characterize the cellular respiration process. Hydrogen peroxide is a poison for the living protoplasm, hence the activity of catalase is of vital importance.” Loew also made the early and important distinction between the substrate (H_2_O_2_) and enzyme in the catalytic reaction at a time when enzymes were only vaguely understood. While peroxidations had been described as early as 1855, the enzymatic nature and biological function of peroxidases would only be investigated much later [11, 20, 21]. The idea of dangerous toxic free radicals formed as by-products of an aerobic metabolism blossomed following the discovery of superoxide dismutase(s) and the demonstrations of superoxide radical actions in biological systems by Irwin Fridovich and his students [14, 15, 22]. Incalculable efforts have been expended to achieve our current understanding of the poisonous and damaging reactions, sources of the radicals and oxidants, as well as the full repertoire of detoxifying enzymes and their roles in physiology and pathophysiology. In addition, novel roles for radicals and oxidants in cell signaling and scavengers in signal modulation were appreciated by the end of the 20th century [23], but are still not fully comprehended.

Indeed, paradigms established by the pioneers of anti-oxidant and free radical-scavenging enzymes guided the discovery of NOD and continue to instruct investigations. Experiments have also been facilitated by a wealth of knowledge, as well as questions waiting for solution, in the areas of Hb structure, distribution, expression, functions, and evolutionary origins [24, 25]. On the other hand, the discovery of NO metabolizing enzymes followed rapidly on the heels of discoveries of the biological production and functions of NO, thus leaving much to discern and discover in a rapidly evolving field. Substantial effort is now being aimed at understanding the biological functions and enzymatic mechanisms of various NODs and the development of NOD-based technologies including research tools, recombinant plants and other organisms, and therapeutic drugs including antibiotics. Novel Hbs continue to be examined for a possible NOD function. In addition, novel non-Hb type NODs and NOD mimetics are under investigation [26].

## 2. Purpose and Scope

 While several reactions of NO with erythrocyte Hb and muscle myoglobin (Mb) including the tight-binding of NO to the ferrous heme, the NO (*Stickstoffoxyd*, *Saltpeterstoffgas*)-mediated oxidation of the oxy-heme, and the formation of NO from nitrite have been under investigation for many decades [27–33], our investigations published in 1998 [34, 35] provided our first glimpse of the ~2 billion-year-old evolutionary link between NO and Hb vis-à-vis the NOD function of *E. coli* flavoHb. The observations offered a distinct and common enzymatic function for the primitive low-abundance Hbs first formally formulated and investigated by David Keilin around 1945 [36, 37], expanded and clarified by Austen Riggs [38, 39] and others [40–42], and pursued early on in the labs of Cyril Appleby [43, 44], Britton Chance [45], Hans Schlegel [46], Dale Webster [47], Jonathan and Beatrice Wittenberg [24], Bärbel Friedrich [48, 49], Robert Poole [50, 51], Austen Riggs [39, 52], Michiko Nakano [53], Robert Poyton [54], Daniel Goldberg [55–57], Keiji Shikama [58–61], Malcolm Potts [62], Robert Hill [63, 64] and many others clearing a wide path to the common NOD function. *However, while appearing attractive to some, the well-known tight-binding of NO to ferrous Hbs *[*K*_*D*_ = ~10 pM]* made the NOD enzyme hypothesis appear rather dubious in the eyes of experts.* Despite this and other challenges, data supporting a primal NOD mechanism-function for diverse members of the ancient Hb superfamily has expanded appreciably in the last 14 years. While the facts that lay obscure or dormant in the pre-1998 literature, and the findings of the first ~7 years have been dutifully and thoroughly reviewed in our 2005 and 2006 publications [65, 66] and elsewhere, my intent here is not only to describe important advances in the area of Hb/NOD research, but also to pose critical questions and discuss new concepts that will help move investigations forward and into new areas. This, of course, has demanded critiques of what I consider lingering misconceptions that continue to plague the field, stymie progress, and lead investigators off the path. It is hoped that the reader finds this paper rigorous, not trite, too pedantic, tame, or polemical. The reader is also directed to more than a dozen recent reviews presenting in-depth perspectives on various aspects of the topic [1, 67–87]. I have attempted to highlight these reviews in pertinent sections of my paper. A distillation and synthesis of the available evidence is crucial since some investigators continue to ponder upon a “common but still poorly defined function of globins” [88]. Here, I thoroughly scrutinize the merits of a common NOD function in the light of the results of more than a decade of research.

## 3. Evolution and Distribution of Hbs

 Several recent phylogenomic studies and reviews have catalogued the broad distribution and lineages of Hbs of various subtypes in the three life kingdoms [1, 89–94]. The amazing array of globins expressed even within individual life forms [3, 42, 74, 95] including humans [96, 97] raise myriad questions that will occupy investigators for many years. Most strikingly, the genomes of most organisms encode multiple Hbs with unknown function. Moreover, relatively few Hbs have been scrutinized for a NOD function. Which sub-types function as NODs? If a life form cannot express a Hb, how does the organism metabolize NO? Why do organisms express multiple Hbs and various Hb sub-types? What primary structure features reliably predict a NOD function and allow annotation of Hb genes? What is the significance of these structural features to function? What are all the possible functions of Hb? And the biggest question—How did Hb function evolve?

 Answers to these many questions have been slow to emerge, but there are fundamental concepts guiding our understanding of Hb functions and evolution. Clearly, numerous proteins possess multiple reactivities and functions [98, 99]; a property that has been colorfully referred to as “moonlighting” [100] or “catalytic promiscuity” [101]. Indeed, those reactivities serve as a framework for protein [globin] evolution. As astutely pointed out by Shikama and coworkers [61, 102], “*Whatever the possible roles of such primitive or ancient globins may be [or might have been], the reversible binding of molecular oxygen to iron[II] must be the primary event to manifest their physiological functions in vivo.*” The fairly unique electronic structure of the ferric heme-superoxo bond in Hbs [103] suggests an even more unique chemical-biological niche for the globins. Thus, while a focus on the evolution of globin structural differences suggests great functional variation [24, 25, 104, 105], a focus on the potentially limited reactivity of the conserved ferric heme-superoxo electronic structure suggests a common function.

 As Max Perutz eloquently stated “Evolution is a brilliant chemist” [106]. Proteins evolve by eliminating old traits and creating new traits from a single protein scaffold. In the case of Hbs, the old traits of flavoHb-NODs include high O_2_ affinities, high autooxidation rates, and internal electron transfer rates that are suited for rapid NO dioxygenation catalysis. New traits include the low O_2_ affinities, low autooxidation rates, external electron donors, lower NO dioxygenation rate constants [65], and a single globin domain that is adapted for O_2_ storage-transport [106]. What is more difficult to fathom and demonstrate is a major O_2_-independent function for Hb that predates the ~2 billion year old, contemporary, and common NOD function [1]. Albeit, NO reductase [69, 107] and electron transfer [108] functions have been proposed for flavoHb and neuroglobin (Ngb), respectively. The discoveries of an estimated 3.8 billion-year-old primordial protoglobin in a “strictly” anaerobic methanogen *Methanosarcina acetivorans* [109, 110] and the single domain “thermoglobin” in *Aquifex aeolicus* (AE000678) [52, 111] provide relic experimental systems to further explore the functional origins of the globin family.

## 4. FlavoHbs, Hbs, and Mbs Function as NO-Metabolizing Enzymes 

 Are Hbs, Mbs, trHbs, and flavoHbs simple NO scavengers or are they highly evolved enzymes? For the two domain flavoHbs, the answer has always been clear. FlavoHbs catalyze NO dioxygenation efficiently, rapidly, and with high fidelity [65, 66]. FlavoHbs belong to the oxidoreductase family utilizing NAD(P)H to incorporate two O-atoms from O_2_ into the substrate NO to form nitrate (2). FlavoHbs catalyzing the reaction are formally called NODs and have been designated EC 1.14.12.17 by the Enzyme Commission of the International Union of Biochemists and Molecular Biologists. Over 4,000 flavoHb protein entries are now labeled primarily as NODs at the US National Institutes of Health web site (http://www.ncbi.nlm.nih.gov/pubmed/). Many more flavoHbs are annotated for a possible NOD function. Since my last listing of flavoHb-NODs in 2005 [65], several flavoHbs notably expressed by human or plant pathogens have been isolated and tested directly for a NOD function. *Aspergillus oryzae*, *A. nidulans,* and *A. fumigatus* express 2 flavoHbs showing a NOD function [112–114]. *Erwinia chrysanthemi* [115], *Giardia intestinalis* [116], *G. lamblia* [117], and *Mycobacterium tuberculosis* [118, 119] flavoHbs have been also investigated. The *E. chrysanthemi* and *Giardia* flavoHbs show appreciable NOD activity *in vitro*

(2) 2NO+2O2+NAD(P)H→2 NO3−+NAD(P)++H+

For many single domain globins (SDGs), an annotation as a NOD has always been less clear. It would appear that all single domain oxy-Hbs catalyze the NO dioxygenation reaction with reported *in vitro* bimolecular rate constants ranging from 10^6^ to >10^9^ s^-1 ^M^−1^ [65, 66, 120–125]. The *Arabidopsis thalania* GLB1 and GLB2 (trHbs) convert NO to nitrate *in vivo* as evidenced by increased NO emissions and decreased tissue nitrate concentrations in globin-deficient mutants [126, 127]. But few SDGs have been shown to couple with a native redox partner for efficient enzymatic NO consumption [128–132]. Moreover, when demonstrations of weak or nonspecific redox coupling have been observed to support turnover *in vitro*, or in heterologous organisms, there has been an understandable reluctance to declare that sufficient evidence for a NOD function [133]. Clearly, *full proof of a NOD function requires the demonstration of catalytic O*_2_ *-dependent NO metabolism within the native organism. Furthermore, a specific electron donor needs to be identified for demonstrations of efficient catalysis in vitro.* To date, only the mammalian Cygb-NOD has been investigated both in cells [134, 135] and reconstituted with a native electron donor (ascorbate or cytochrome b_5_) *in vitro* [135, 136]. In addition, many SDGs have shown evidence of a protective enzymatic NOD function including muscle Mb [137], *Synechococcus* truncated Hb [138], *Synechocystis* cyanoglobin [133], and the dual function nitrate reductase-fused trHb in raphidophytes [139]. However, one cannot declare a NOD function a priori. A NOD function requires a demonstration of enzymatic turnover. Protective effects of SDGs could also be attributed to nonenzymatic, rapid, and competing NO oxidation (dioxygenation) and nitrosylation reactions of Hbs similar to those of erythrocyte Hb and muscle Mb [31, 32, 140]. This is a small distinction, but a potentially important one.

## 5. Myriad NO Functions: Bioenergetic Intermediate, N Source, Toxin, and Signal Molecule

 To understand the physiological functions of NODs (Hbs), knowledge of the burgeoning field of NO biology is required. NO is fairly ubiquitous and serves important roles in bioenergetic transformations, immunity, and signaling in diverse life forms. Quantitative knowledge of the sources of NO, target reactions of NO, and localized steady-state NO levels is also required to discern the competing and overlapping roles of NO. Moreover, within specific cells and tissues, these factors vary in complex ways [198, 199]. Nevertheless, instructive generalizations can be made.

 In microbes, algae, and plants, NO serves as an important role as a bioenergetic intermediate and nitrogen source. The process of anaerobic or microaerobic denitrification, particularly in soil microorganisms, generates NO as an obligate intermediate in the energy-yielding reductive dissimilation of NO_3_^−^ to N_2_ [17, 200]. Nitrate reductases generate nitrite, and nitrite reductases generate NO for reduction by proton-motive force generating membrane-bound NO reductases and N_2_O reductases. FlavoHb (NOD) can recycle the denitrification intermediate NO to NO_3_^−^ and is required for optimal microaerobic denitrification, and presumably energy production, by fungi [200] and bacteria [48]. NO is also an important intermediate in the energy-yielding pathway of microaerobic ammonia oxidation or nitrification [201, 202]. Alternatively, NO can be generated through non-specific reduction of nitrite by nitrate reductases found abundantly in plants, algae, and microbes [203–205]. Neighboring soil microorganisms and plants can assimilate N through the Hb-catalyzed dioxygenation of diffusible NO to NO_3_^−^. In addition, NO_3_^−^ generated *via* catalytic NO dioxygenation can provide an electron sink for fermentative energy production by plants [206], bacteria, and fungi [52, 200].

 NO is also an important intermediate in the Earth's nitrogen cycle [207, 208]. Anthropogenic sources, such as N-fertilization, NO_*x*_ pollution, and waste generation, may contribute to excess formation of NO and the greenhouse gas N_2_O [207]. Rhizobial NODs serve an important role in converting NO to NO_3_^−^ in plants and in doing so avert N_2_O formation by rhizobia and soil microbe NO reductases [87]. Indeed, *Pseudomonas stutzeri* engineered for flavoHb overproduction emit less N_2_O during microaerobic denitrification [209]. Photoilluminated leaf chloroplast nitrite reductase is also an important source of atmospheric N_2_O presumably *via* the reduction of assimilated NO_2_^−^ to NO [210], and NO to NO^−^ followed by the combination of 2 NO^−^ to form N_2_O. In this case, a chloroplast NOD (trHb) may serve as an important geochemical function by decreasing NO levels and N_2_O formation.

*NO acts as a toxin and signal molecule throughout the biosphere*. In addition to the numerous sources of NO described above, NO is produced by nitric-oxide synthases (NOSs) and various nitrite-reducing activities in many different organisms including humans [211]. Removal of NO by catalytic NO dioxygenation can thus serve to prevent NO toxicity or attenuate NO signaling. Understanding NO toxicity and signaling in the biosphere requires an understanding of the many reactions of NO as well as its concentrations. I have listed only a few of the important biochemical reactions of NO and the relevant biological functions or consequences in Table 1. The representative list of reactions clarifies why NO is an outstanding natural antibiotic and antitumor agent and why NO metabolism by NODs or NORs provide a direct path for cellular resistance. The toxicology of NO has been discussed in greater detail in an excellent recent review by Toledo Jr. and Augusto [189]. *Not too surprisingly, many of the toxic reactions of NO have been exploited by Nature for NO signaling functions.* These bifunctional toxic reactions include ferrous heme nitrosylation (e.g., soluble guanylate cyclase activation), iron-sulfur center disruption (NsrR and IRE-BP) [193], mononuclear iron-binding (e.g., prolyl hydroxylase, NorR and ACO), and the NO/bound O_2_^−^ reaction (e.g., DevS, DosT and FixL).

 When evaluating the competitive reactions of NO in complex systems, it is very valuable to know steady-state concentrations of reactants and bimolecular rate constants. These values allow us to make some simple and powerful calculations. For example, we can determine the rate of NO removal and the maximum flux of NO going to toxic peroxynitrite formation in a cell containing O_2_^−^ and oxy-Hb. If the steady-state [O_2_^−^] = 10 pM, [NO] = 0.1 *μ*M, and [oxy-Hb] = 0, then the rate of NO removal and peroxynitrite formation = *k*_2_ [NO] [O_2_^−^] = 7 nM NO s^−1^, where *k*_2_ = 6.9 × 10^9^ s^−1^ M^−1^. If oxy-Hb is also present at 1 *μ*M, and its bimolecular rate constant (*k*_3_) for reaction with NO = 3 × 10^7^ s^−1^ M^−1^, then the rate of NO removal = *k*_2_ [NO][O_2_^−^] + *k*_3_ [NO][oxy-Hb] = 30,007 nM NO s^−1^. We can also see from the relationship that at any steady-state [NO] the ratio of the NO flux to O_2_^−^ and oxy-Hb equals *k*_2_[O_2_^−^]/*k*_3_[oxy-Hb], or in this case 0.0023. Thus, under these physiologically relevant concentrations, only 0.23% of the NO will escape detoxification by oxy-Hb and form toxic peroxynitrite. Similar approximations can be made for other competing reactants.

## 6. FlavoHbs and Hbs Detoxify NO

 A large number of flavoHbs and Hbs have been shown to detoxify NO, and many of these have been listed and described in my 2005 review [65] and in the recent Forrester and Foster review [67]. The list of organisms that are presumed to detoxify NO using flavoHbs and Hbs has expanded profoundly with the increase in genome sequencing and *in silico* analysis. Not too surprisingly, far fewer experimental demonstrations of organisms utilizing flavoHbs or Hbs for their protection against NO have been reported. Recent experiments support NOD functions for flavoHbs or Hbs expressed by *Bacillus subtilis* [212, 213], *Staphylococcus aureus* [214, 215], *Aspergillus oryzae* [112, 216], *A. nidulans* [113], *A. fumigatus* [114], *Yersinia pestis* [217], *Vibrio fischeri* [218], *Pseudoalteromonas haloplanktis* [219], *Campylobacter jejuni* [220, 221], the Japanese shrub *Alnus firma* [182], *Sinorhizobium meliloti* [183], *Botrytis cinerea* [222], and *Synechococcus* [138]. Expression of *Mycobacterium leprae* GlbO [223] and *Synechocystis Syn*Hb [133] alleviates NO toxicity in *E. coli* supporting a NOD function. And expression of cotton non-symbiotic Hb in Arabidopsis seedlings conferred resistance to NO [224]. Furthermore, novel or special interest Hbs including the mammalian Cygb [134–136], and the algal raphidophyte *Heterosigma akashiwo* nitrate reductase fused with a trHb (NR2-2/2HbN) have been characterized as protective NO-metabolizing and NO-detoxifying enzymes [139]. Interestingly, the *Mycobacterium tuberculosis* flavoHb (Rv0385) reportedly showed little NOD activity *in vitro* [119] and provided negligible nitrosative stress protection to Hmp-deficient *E. coli* [118]. Similar functional uncertainty exists for the NO-inducible truncated Ctb from *C. jejuni* [225–227]. *With the advent of rapid genome sequencing, annotation of various flavoHbs and Hbs as NODs *(*EC 1.14.12.17*)* in genome databases has become prodigious. What is needed are more reliable criteria for annotating a NOD function.*

## 7. NOD Functions for FlavoHbs and Hbs in Microbial Pathogenesis

 NO is produced as a natural antibiotic and antitumor agent in the innate immune response of animals and plants. Microbes and tumor cells have the capacity to resist NO toxicity [228]. Microarray analyses of mRNA [152, 183, 213, 214, 220, 229–238] and *in silico* reconstruction of transcription networks [152, 233, 239–241] in microbes and mammalian cells have revealed myriad adaptive changes potentially protecting against NO toxicity during microbial pathogenesis and inflammation. NODs, NORs [17, 151, 242], Fe-S cluster [243], and DNA repair enzymes [243], heme biosynthetic pathways [113], NO-resistant metabolic pathways (e.g., glucose metabolism and respiratory oxidases) [159–161, 240, 241], nitrite metabolism [213], and components of iron uptake systems form important and common elements of the nitrosative stress defense. Remarkably, in plants [141], *Vibrio fischeri* [162], and the fungal pathogen *Candida albicans* [238], NO poisoning of respiration is apparently averted by the induction of NO-resistant alternative oxidase. Mycobacteria survive the hazardous environment of macrohages with GlbN (trHbN), GlbO (trHbO), and heat shock protein GroEL2 induction with GlbO providing greater protection than GlbN within macrophages [244]. Some aerobic organisms do not utilize a flavoHb-NOD, but apparently utilize sole NORs for NO metabolism [232], but these are the exception rather than the rule.

 The accumulated evidence supports the *hypothesis that NO metabolism and detoxification by NODs and/or NORs form the first, and most critical, line of defense against NO toxicity in microbes.* However, results reviewed in the pre-2005 literature revealed only a modest advantage of the inducible flavoHbs and Hbs for microbes in the chosen infection models [65]. Tail vein injections of NOD-deficient *Candida albicans* showed limited effects on mouse survival [95, 238, 245], and those effects appeared NOS-independent [238]. Inhalation of *Cryptococcus neoformans* in mice showed similar modest extensions of mouse survival times with a flavoHb deficiency [246]. The effects of HmpX (flavoHb) deletions in *Erwinia chrysanthemi* infections of *Saintpaulia ionantha* (African violet) plants were more impressive, but interpretations were subject to potential effects of deletions of neighboring pectate lyase virulence genes [115, 247]. Nevertheless, Boccara et al. convincingly argued an important role for HmpX in the pathogenesis of *E. chrysanthemi*. Not only does HmpX (NOD) protect against NO toxicity, it also impacts the NO-regulated hypersensitive response required for plant immunity [115, 224, 248]. Clearly, *more and better models of microbial infection are required to discover the full involvement of flavoHbs and Hbs in microbial pathogenesis, albeit the prospects appear limitless*. For example, mucosal infections by *C. albicans* are far more common and applicable than blood stream infections. Most experimental designs also ignore the effects of the metabolic state of microbes upon subsequent infectivity [249]. For example, naïve and metabolically depleted *E. coli* are more susceptible to NO toxicity and bacteriostasis than actively growing bacteria [35]. More impressive protective roles for the flavoHb-NOD in rodent models of *Salmonella typhimurium* [250], *Staphylococcus aureus* [214], uropathogenic *E. coli* [251], *Yersinia pestis* [217], *Vibrio cholerae* [252] infections and in squid models of *Vibrio fischeri* symbiosis [218] have been subsequently reported. Interestingly, in the intestinal model of *V. cholerae* infection, flavoHb (Hmp) was more critical for virulence than the NO defense regulator. Stern and coworkers suggest that low constitutive levels of flavoHb protect *V. cholerae* from NO-dependent elimination [252]. In the case of the plant fungal necrotroph *Botrytis cinerea*, flavoHb-NOD was not found to be a virulence factor [222].

## 8. Critical Roles for Hb-NODs in Preserving Bioenergetics

 Hbs serve two most vital roles in the production of energy *via* oxidative-phosphorylation. When oxygenated, Hbs can prevent NO poisoning of the citric acid cycle enzyme aconitase, heme-copper terminal oxidases, and respiration by catalyzing NO dioxygenation [35, 163]. NO poisoning of terminal oxidases and respiration is particulary problematic during hypoxia because of the strong competition between NO and O_2_ [~1 : 1000] for binding the binuclear heme a_3_/Cu*_B_* site and the noncompetitive binding of NO to the oxidized Cu*_B_* site [159, 160, 164]. If sufficiently abundant, and having a low affinity for O_2_ binding, Hbs can also store and release O_2_ to sustain respiration during transient episodes of hypoxia. These two functions, or NOD activity alone, can explain the strong protection nerve Hbs afford nerve excitatory activity during episodes of anoxia/hypoxia [253, 254], muscle Mb provides the heart during hypoxia [137, 255], Cygb affords fibroblast respiration [134], flavoHb affords *E. coli* respiration [163], *Vitreoscilla* Hb affords either *E. coli*, plants or zebrafish (*Danio rerio*) engineered for biotechnological applications [78, 256–258], nonsymbiotic Hb expression has on maize ATP levels and energy charge during hypoxia [63, 74], and legume Hb affords *Rhodobacter japonicum* respiration [43] and soybean nodule bacteroid N_2_ fixation [125, 259]. The inducible *Sinorhizobium meliloti* flavoHb plays a similar role in protecting alfalfa bacteroid N_2_ fixation and fostering symbiosis [183, 260]. *These strong dual effects on organism energetics *(*and survival*)* may have constituted the earliest, most powerful, selective force and circumstance for Hb to evolve from an efficient low-level expression NO dioxygenase to a highly expressed O*_2_ *storage-transport protein.* Ironically, the existence of Antarctic icefish lacking in Mb and Hb also supports the notion that these proteins evolved for dual functions. However, in this case, the loss O_2_ transport-storage and NOD functions are apparently compensated for by a NO-augmented tissue vascularization, luminal diameters of blood vessels, and mitochondrial densities [261].

 From a different perspective, the product of the NOD reaction, nitrate, can increase glycolytic ATP production by microbes and plants during the transition from the hypoxic to anoxic state by serving as an oxidant reservoir for NADH oxidation *via* nitrate reductases and nitrite reductases [52, 206].

## 9. Beyond NO Detoxification: Signaling

 The ability of Hb to consume NO in the mammalian vasculature [262, 263] and Mb to decompose NO in muscle [137, 255, 264] has been an important issue since the conception of the role of NO in controlling blood flow. Indeed, the abundance of red blood cell Hb and myocyte Mb prompted the consideration of mechanisms that would be able to preserve NO in the vasculature or muscle tissues [265] in the face of certain destruction. These mechanisms include NO diffusion barriers [266–268], Hb/Mb-mediated NO sequestration and release [140] and, more recently, Mb or Hb-mediated NO formation from NO-derived nitrite [269]. The rapid reactions of NO with oxy and deoxy Hb and Mb also led to the early view that these reactions would impair their O_2_ transport-storage functions [31, 270]. However, the role of Hbs in modulating spatial and temporal NO signaling is subtle and slowly becoming apparent. In tissues, Hb, Mb, Ngb and Cygb may act as catalytic NO sinks that together with NOS dynamically and spatially determine steady-state NO levels, soluble guanylate cyclase (sGC) activation, and myriad signaling actions [198, 199, 255, 262, 271, 272] including regulating cerebral blood flow, synaptic efficiency and neurotransmitter release [273].

 Griffiths and Garthwaite [271] formulated a “clamp” model for a better understanding of the consequences of NO consumption on NO steady-state levels and NO signaling functions in mammalian tissues. In this model, the sink (i.e., NOD) translates different rates of NO formation with a tissue volume into proportional steady-state NO concentrations and clamp [NO]. The NOD thus serves to “amplitude-code” NO signals. The NO inactivation rate also governs the rates of rise and fall of NO concentrations as NO sources switch on and off. In target cells, activation of sGC then causes cGMP to accumulate rapidly to levels that are graded with the prevailing NO concentration. A high NO inactivation rate endows the NO signal with temporal meaning. In addition, NO sinks add a spatial dimension for NO signals and provide additional mechanisms for regulating NO signaling such as *via* cellular O_2_ concentrations [142, 274, 275]. Furthermore, physiological inactivation mechanisms for signaling molecules generally have properties that are tuned to those of the receptors. In other words, a NOD needs to modulate NO steady-state levels at the 0.1–10 nM concentration levels that modulate sGC activity *in vitro* [191, 192]. Using quantitative real-time recording and modeling of neuronal NO signals *via* sGC activation in phosphodiesterase-deficient cells, Wood et al. [276] recently extended the estimates of steady-state [NO] in neuronal tissues to 0.25–3 nM with rates of NO generation estimated at 0.036 to 0.360 *μ*M NO s^−1^.

 Chen and Popel [277, 278] have mathematically modeled steady-state NO production rates in vascular and perivascular tissues and estimated similar values ranging from 0.017 to 1.5 *μ*M NO s^−1^ that are dependent upon concentrations of the NOS isoforms and tissue O_2_ concentrations. Endothelial NOS (NOS3) expressed at lower concentrations (0.045 *μ*M) produces lower NO fluxes whereas neuronal NOS (NOS1) is expressed at higher levels (0.3–0.9 *μ*M) and produces larger NO fluxes. In these studies, estimates of NO steady-state concentrations in the perivascular tissue ranged from 0.3 to 51 nM and were O_2_ dependent. These values for [NO] are more than an order of magnitude lower than previous estimates. Importantly, the resulting steady-state NO concentrations are well within the range required for sGC activation (0.1–10 nM) [191, 192, 272]. The authors of these investigations made a number of simplifying assumptions including a rate of tissue NO consumption that was linearly proportional to NO and O_2_ concentrations as expressed by the bimolecular rate equation, rate = *k*_*t*_ [NO][O_2_][cells], where *k*_*t*_ = 5.4 × 10^−10^ *μ*M^−1^ s^−1^ [cells/mL]^−1^ and [cells] = 10^8^ cells mL^−1^ [275]. However, the liver parenchymal cell activity used for the estimation of “*k*_*t*_” may be low since mammalian cells express NO consumption activity levels ranging from 2 to 20 nmol NO min^−1^10^7^ cells^−1^. Using a cell concentration estimate of 10^8^ cells mL^−1^, NO metabolic rates of 0.17 to 1.7 *μ*M NO s^−1^ corresponding to *k*_*t*_ values of 5.1 × 10^−10^ to 5.1 × 10^−9^ *μ*M^−1^ s^−1^ [cells/mL]^−1^ are calculated. A larger NO consumption activity and *k*_*t*_ value lowers the steady-state NO concentration estimates for the vascular and perivascular tissues. Moreover, contrary to the model, the activity shows normal Michaelis-Menten enzyme kinetics with *K*_*m*_(NO) and *K*_*m*_(O_2_) values of 0.2 *μ*M and 17 *μ*M, respectively [142], thus further complicating estimates of steady-state NO levels.

**Figure 1:**
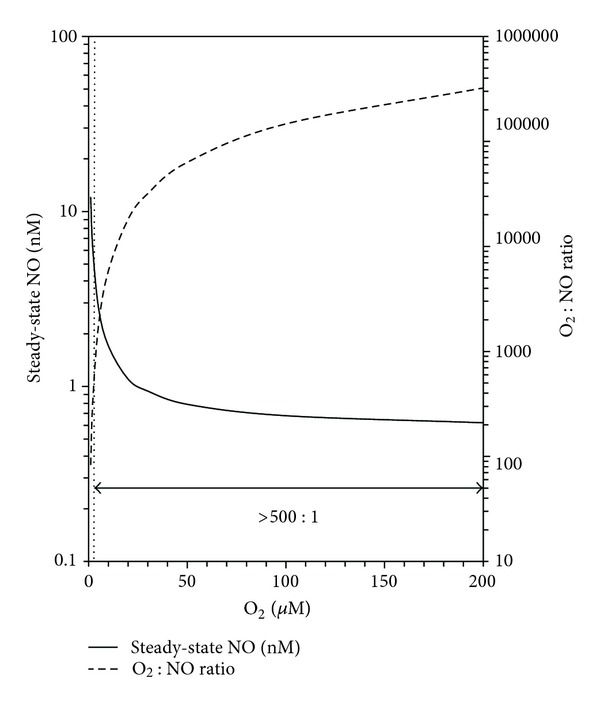
Modeling steady-state [NO] as a function of O_2_ concentration with Cygb-NOD catalysis at a constant NO flux. The steady-state [NO] decreases with a higher Cygb-NOD turnover at higher [O_2_] (*solid line*). At low [O_2_], an elevated steady-state [NO] decreases the O_2_ : NO ratio (*dashed line*), and at O_2_ : NO ratios <500, NO inhibits Cygb-NOD [135]. A higher [Cygb], or lower NO synthesis rate, would be required for Cygb-NOD to maintain an O_2_ : NO ratio of >500 : 1 at a lower [O_2_]. Calculations were for 1 *µ*M Cygb, *k'*_NOD_ = 3 × 10^7^ s^−1^ M^−1^, and a NO synthesis rate of 1.7 *µ*M NO s^−1^. Calculations were simplified by applying the experimentally measured *K*_*m*_(O_2_) = 20 *µ*M to derive a *k'*_NOD_ apparent = *k'*_NOD_ [O_2_]/*K*_*m*_(O_2_) + [O_2_] for the various O_2_ concentrations. The graded NO inhibition of Cygb-NOD is reflected in the apparent *K*_*m*_(O_2_), and the effect of O_2_ on NO synthesis rates are ignored.

 Modeling of NO levels in tissues can provide quantitative and qualitative insights into the roles of potential NO consumption pathways. For example, for superoxide radical to act as a bimolecular pathway for NO removal at rates of 0.17 to 1.7 *μ*M NO s^−1^, steady-state O_2_^−^ levels of 140 pM to 1.4 nM would be required. These concentrations are well beyond the ~8 pM O_2_^−^ estimated within O_2_^−^-generating mitochondria [279] and far greater than the level expected in the cytosol. On the other hand, for a Hb-NOD, like Cygb-NOD with a maximal turnover rate of ~1.2 s^−1^ [135], to act as the sole catalyst, a tissue Cygb concentration of 0.14 to 1.4 *μ*M would be required. These concentrations are within range of the globin concentrations typically seen in nonerythroid cells [4, 280–282] and plants [74, 283]. Moreover, an O_2_-dependent NO consumption activity provides a feedback mechanism for controlling O_2_ delivery to hypoxic tissues *via* decreased NO consumption, [NO] elevation, sGC activation, smooth muscle relaxation, and increased capillary blood flow [142, 255, 262]. Halligan et al. [134] and Liu et al. [136] recently provided evidence and arguments supporting a role for Cygb-NOD in controlling NO levels and vasorelaxation. Similar roles for Mb expressed in smooth muscle [4, 137] and oxidase-generated O_2_^−^ [284] have been previously suggested. *Various Hb-NODs are excellent candidates for the long sought dynamic sensor-regulator controlling tissue O*_2_ *delivery and pO*_2_. The plots in Figure 1 illustrate the potential for Cygb, or other globins, to act as O_2_-dependent modulators of NO steady-state levels within the physiologically relevant parameters. Caution is warranted since this simple model assumes that the globin is the major catalyst. Cells may express multiple NO dioxygenation catalysts [135].

 Cytosolic globins have the potential to influence NO signaling and cell phenotypes through NO scavenging; however, enzymatic NO scavenging by cytosolic mammalian globins has not been demonstrated in most cells to date (e.g., see [285]). Cygb is the exception [134, 135]. In mammals, *α* and/or *β*-chains of the red blood cell HbA_0_ have been detected in many different cells including hepatocytes [286], neurons and glial cells [287–291], macrophages [285], alveolar type II epithelial lung cells [282, 292], and mesangial kidney cells [293] suggesting additional Hb functions. Hb is enriched in pyramidal hippocampus and parietal grey matter neurons of Alzheimer's patients [294]. Other studies have demonstrated the expression of HbA_0_ *α*- and *β*-chain mRNA in non-erythroid cells. Visceral metastases of breast carcinoma express elevated Hb *β*-chain [295]. Cygb and Ngb are expressed in a variety of animal cells and tissues including neurons [296]. In cancer, tumor growth is suppressed by expressing *E. coli* flavoHb-NOD in brain gliomas [297], overexpressing Cygb in head and neck squamous cell carcinomas [298, 299] or, paradoxically, by decreasing Mb in breast cancer cells [7]. *Globins, acting as NODs, may either decrease the NO signal eliciting a metastatic cell phenotype or increase NO detoxification and the resistance of tumor cell targets to endogenously generated NO *(see Table 1). Removal of NO would decrease NO-elicited hypoxic tissue vascularization, mitochondrial biogenesis [300, 301], and the Hif-1*α*-orchestrated gene array expression important for bolstering hypoxic metabolism and the metastatic phenotype. The prolyl hydroxylase controlling Hif-1*α* stability and function is sensitive to inhibition by NO [170]. NO induces and stabilizes Hif-1*α* and bolsters the hypoxic adaptation of cells [302]. Moreover, Hif-1*α* upregulates Mb and Ngb expression [7, 9], thus providing a potential feedback loop for [NO] homeostasis. In this model, increased NO removal would decrease prolyl hydroxylase inhibition, destabilize Hif-1*α*, and attenuate globin expression and NO removal.

 Globin-regulated NO signaling may also be important for the normal functioning of neurons. Expression of HbA_0_ in dopaminergic neurons is linked with pathways involved in O_2_ homeostasis, oxidative stress, iron metabolism, NO synthesis, and oxidative phosphorylation [287]. Hif-1*α* expression was decreased in HbA_0_ expressing neurons and targets of Hif-1*α* were altered [287]. Ngb deficiency in mouse brain exacerbates the Hif-1*α*-regulated response to hypoxia [303] further suggesting effects of the neuronal Ngb on [NO] and NO on Hif-1*α* stability.

 There is also evidence for (flavo)Hbs modulating NO signaling pathways in fungi and bacteria. FlavoHb expression affects *Dictyostelium discoideum* development [304], and *Aspergillus nidulans* sexual development and mycotoxin production [305]. NO scavenging by flavoHb attenuates the expression of the nitrosative stress response (e.g.,* norVW*) [151], affects the swarming behavior of *E. coli* [306], and maintains squid-*Vibrio fischeri* [218] and *Medicago truncatula-Sinorhizobium meliloti* [260] symbioses.

 R. Hill recently published an up-to-date and thorough review discussing the many known and potential roles for Hbs in modulating NO signaling processes in plants [82]. For example, the *Arabidopsis* Hb, GLB1, modulates salicylate, ethylene, and jasmonic acid responses to *Pseudomonas syringae* and *Botrytis cinerea* infections [307]. Lowering GLB1 levels and increasing NO levels elevates the defense against pathogens *via* multiple hormones. GLB1 expression also decreases ethylene-induced upward leaf movements in response to root hypoxia (hyponasty) [126].

## 10. Regulation of Globin Expression and NOD Function

 A variety of mechanisms exist for controlling the expression of flavoHbs and Hbs in different organisms. Numerous organisms are now known to regulate globin expression in response to NO and hypoxia [65, 80] *via* specific NO and O_2_-responsive transcription factors as part of NO and hypoxic stress responses [67, 80]. Other inducing signals emanate from the inhibition of the electron transport chain [54], iron deficiency, oxidative stress, osmotic stress, cold stress, or hormones. A complex interplay of NO, O_2_, temperature [237, 308], iron availability, nitrate, nitrite, and oxidative stress can control Hb-NOD expression in various organisms. Moreover, *it is impossible to deduce a NO detoxification function of a protein/gene solely from inducing signals since many NO-inducible proteins/genes are accessory *[183]* and not all NO detoxifying enzyme *(*NOD*)* genes are responsive to NO *[95, 128, 142, 309, 310].* Nevertheless, given the common capacity of Hbs to function as NODs, the induction of a Hb by NO should be considered strong evidence for a NO metabolic function.* It is also important to remember that NO can be produced from media nitrate and/or nitrite and that high levels of apparently constitutive (flavo)Hb expression [95, 138, 310] may be due to endogenous NO formation.

 An impressive literature has amassed describing NO-responsive regulation of globin expression in various life forms since the writing of my 2005 review [65]. At that time, a role for the NO-sensitive regulator NorR in the context of the transcriptional regulation of *norVW* in *E. coli* [151, 194, 311] and *norBC* in *Ralstonia eutropha* [312] was known. The mechanism of NO-sensing by NorR was subsequently elucidated by D'Autréaux et al. [313]. Roles for the ferric uptake regulator (Fur), methionine repressor (MetR), superoxide response regulator (SoxRS), fumarate nitrate reductase regulator (Fnr) in *E. coli*, or *Salmonella hmp* (flavoHb) transcription had also been investigated and reported [69, 314–316], but those regulators did not satisfactorily explain NO induction [229, 317–319]. The advent of genomics and systematic and comparative operon analyses [239] greatly accelerated the identification NO-sensing regulators and the characterization of globin regulation in various bacteria. Stephen Spiro recently reviewed the literature on NO sensor regulators in the larger context of gas sensors in bacterial nitrogen metabolism [80]. Efforts to identify NO-sensing regulators in representative yeast and fungi have progressed more slowly. Upregulation of globin expression in plants, algae, and animals in response to NO and hypoxia have also been described and is consistent with a NOD function. Here, I briefly review the NO and hypoxia sensing regulators of globin expression in the context of the putative NOD function.

### 10.1. Bacteria

 NO-regulated NORs and NODs cooperate in the NO defense in bacteria. However, within any single organism, NOD and NOR appear, for the most part, to be under the control of different NO sensor regulators. On the other hand, homologous regulators control either NOR or NOD in different organisms. For example, the sigma-54 dependent transcription activator NorR regulates NOR expression in *Ralstonia eutropha* [312] and *E. coli* [151, 194, 311] in response to nanomolar NO, but NorR regulates NOD (*hmp*) expression in *Vibrio cholera* [239, 252]. Similarly, in *Pseudomonas aeruginosa*, rpoN, a sigma-54 homologue, and FhpR, a NorR homologue, regulate *fhp* (flavoHb) in response to NO [320]. Through a veritable *tour de force*, S. Spiro and coworkers demonstrated that NorR utilizes an EPR-detectable mononuclear iron center embedded in a PAS-GAF domain to bind and sense NO and to activate transcription of *norVW* in *E. coli* [313]. It is this iron center that can also react with peroxide to impair the NO response [321].

 In *Salmonella* [250], *E. coli* [80, 322], *B. subtilis* [213, 323] and many other bacteria, the transcription repressor NsrR senses NO *via* a labile [4Fe-4S] center containing a solvent-exposed NO-reactive iron atom [195, 322]. The NO sensing mechanism appears similar to the mechanism of iron-sulfur dehydratase inactivation by NO, O_2_^−^ or iron chelators [143, 144, 324]. Curiously, early investigators reported *E. coli* flavoHb upregulation following exposure to the O_2_^−^-generating redox-cycling agent paraquat [317] and the ferrous iron-chelator dipyridyl [50], effects presumably mediated *via* NsrR Fe-S center destruction [322]. In *Campylobacter*, NssR, a Crp-Fnr superfamily member, controls expression of the Cgb-NOD and trHb Ctb, presumably through NO interactions with its iron-sulfur center [220, 227]. N_2_-fixing Sinorhizobium utilizes NnrR and FixLJ to sense NO [183] and upregulates transcription of >100 genes including *hmp*. FixL contains heme and can potentially sense NO *via* nitrosylation and dioxygenation reactions and upregulates NOD (*hmp*) [183]. NnrR is also a Crp-Fnr superfamily member and upregulates NOR in Sinorhizobium [183], and nitrite reduction in the ammonia-oxidizing NO-generating lithoautotroph *Nitrosomonas europaea* [201, 325]. Discoveries of additional NO sensor-regulators in bacteria are anticipated. 

 Many bacteria have long been known to upregulate (flavo)Hb expression in response to hypoxia [46, 50, 53, 326]. The common regulator appears to be Fnr, and the signal is O_2_, not NO [309]. Induction of a NOD in response to hypoxia makes sense since O_2_ is a cosubstrate for the NOD reaction and since NO is more toxic to respiration at a low pO_2_.

### 10.2. Yeast/Fungi

 Progress has also been made in understanding NO regulation of flavoHbs in fungi and yeast. In *Saccharomyces cerevisiae*, the Fzf1p regulator is required for NO induction of *YHB* transcription [236]. In *Candida albicans*, CTA4 a zinc-finger protein is required for induction of *YHB1* transcription [245], and Cwt1p acts as a repressor of a nitrosative stress regulon that includes *YHB1* [241]. NO upregulates flavoHb (NOD) expression by *Aspergillus oryzae* [216], the plant pathogen *Botrytis cinerea* [222] and other fungi [65] presumably *via* similar mechanisms. The full details of NO sensing and transcription activation remain to be elucidated.

 One of the more interesting discoveries has been that the *Fusarium graminearum* virus-DK21 downregulates flavoHb mRNA and protein expression by the pathogenic plant fungus *Fusarium graminearum* along with virulence [327]. A role for the flavoHb in virulence is suggested.

### 10.3. Photosynthetic Organisms

 Numerous examples of NO and hypoxia upregulating Hbs in plants have been described and reviewed [74, 81, 82, 328]. The fused nitrate reductase-trHb (NR2-2/2HbN) is induced by NO in the microalgal species *Heterosigma akashiwo* [139]. Arabidopsis class 1 nsHb is induced during growth with nitrate [329]. Two nonsymbiotic trHb genes are strongly induced by nitrate, nitrite, and NO in cultured rice cells [330]. Cotton and wheat nsHbs are induced by NO [224] or NO donors [331]. Only one of the five Hb genes, LjHb1 encoding a nsHb, is induced by NO and hypoxia in *Lotus japonicum* [308, 332]. Another nsHb (LjHb2) is induced by sucrose, abscisic acid, and osmotic stress. Clearly, not every Hb with a capacity for a NOD function is induced by NO or hypoxia. For example, the cyanobacterium *Synechococcus* sp. GlbN provides resistance to NO, but is apparently not inducible by NO, nitrate, or nitrite [138].

### 10.4. Protists

 The protective *Giardia lamblia* flavoHb-NOD is induced by nitrite, nitrosoglutathione, and NO donors [116].

### 10.5. Animals/Humans

 Numerous animal Hbs are reportedly induced by hypoxia, but apparently none by NO. The Hb *β*-chain is induced in macrophages by interferon and lipopolysaccharide [285] which also elicit NO synthesis. However, the induced Hb *β*-chain failed to increase NO consumption by macrophages [285]. HbA_0_ chains are expressed in rat ischaemic rat neurons [333] and are upregulated in response to hemorrhage [291], hemin, erythropoeitin [289], and oxygen-glucose deprivation [333]. The chains are not always coordinately regulated; oxygen-glucose deprivation increases rat neuronal *α*-chain mRNA 1.9-fold, but decreases Hb *β*-chain mRNA 3-fold [333]. HbA_0_ is also reportedly present in hippocampal and parietal grey matter neurons of Alzheimer's patients [294], a hypoxic and inflammatory condition that may induce globin expression. Hb *α*- and *β*-chains are also expressed in alveolar type II lung epithelial cells in response to hypoxia [282, 292] and Hif-2*α* or Hif-1*α* control transcription [334]. Ngb is induced by hypoxia [335] and is under control of the master transcriptional regulator of the hypoxic response, Hif-1*α* [336–338]. Hypoxia also induces fish Mb in nonmuscle cells including liver, gill, and brain [5]. Numerous other studies have shown elevated expression of mRNAs for Hb *α*- and *β*-chains, Mb, Cygb, and Ngb in non-erythroid cells and especially hypoxic tumors [7, 9]. What remains unclear is whether these animal globins have a significant capacity for NO dioxygenation and modulation of NO functions and how this relates to pathophysiology. For example, Ngb-deficiency in mice leads to changes in Hif-1*α*-regulated pathways in response to hypoxia [303]. Is this due to deficient NO metabolism and NO stabilization of Hif-1*αvia* prolyl hydroxylase inhibition? Is NO, like O_2_, a signal that normally controls Hif-1*α*?

 While knowledge of the many factors and mechanisms controlling transcription or translation of globins in various life forms is revealing and often supportive of a NOD function, a quantitative knowledge of the NO and O_2_ concentration dependencies of the responses is critical for understanding Hb function in NO homeostasis control, detoxification, and/or signal modulation. The overriding question is: What level of NO is normal and how do cells maintain and respond to changes in the concentration of NO *via* globin synthesis regulation?

## 11. Hb Structure(s) and the NOD Mechanism

*The molecular, atomic, and electronic details of the NO dioxygenation reaction form the heart of our understanding of Hb structure-function and evolution. Proposals of a common, intrinsic, and ancient NOD function require a thorough understanding of the reaction and the chemical properties of Hbs or flavoHbs that make them either good or poor catalysts*. There are several recognized basic requirements for a NOD function that Hbs must possess. These include

1. a high O_2_ affinity,
2. a mechanism for decreasing NO binding to heme,
3. a superoxide radical-like character of the bound O_2_,
4. a protected pocket for the peroxynitrite intermediate,
5. an O-atom isomerization mechanism,
6. a mechanism for nitrate egress,
7. a mechanism for univalent reduction.

Here, I will focus on new knowledge, key concepts, and current questions about Hb chemistry that are directly related to the NO dioxygenation reaction, NOD catalysis, and a NOD function. New investigators of Hb-NODs should consult the seminal literature [34, 49, 52, 339–341] and prior reviews describing (flavo)Hb structure-function-evolution [1, 65–67, 71, 72, 102, 342] for background and additional information. Mowat et al. provide an excellent recent overview of the flavoHb-NOD structure-function [72].

### 11.1. High O_2_ Affinity

(3) HbFe2++O2⇌HbFe2+(O2)

By definition, all Hbs bind O_2_ reversibly (3). However, Hb affinities for O_2_ vary greatly with equilibrium dissociation constants (*K*_*D*_ values) ranging from low nM to greater than 20 *μ*M O_2_. On- and off-rates for O_2_ determine O_2_ affinities, and these are influenced by structure as described in an excellent recent review by Marti et al. [343]. A high O_2_ affinity is critical for limiting NO inhibition during catalytic NO dioxygenation. Mutations increasing the O_2_ off rate of flavoHb increase NO inhibition [341]. O_2_ must be able to outcompete NO for binding the ferrous heme for a Hb to function as a NOD. Hbs with lower O_2_ affinities (e.g., Cygb) function as effective NODs but only at a low [NO] relative to [O_2_] [135].

### 11.2. Mechanisms for Decreasing NO Binding to Heme

 Competition between NO and O_2_ for binding the ferrous heme, as predicted from transient kinetic measurements, should prohibit a NOD function for most (flavo)Hbs [65, 344]. For many Hbs, the *K*_*D*_(NO) value determined by laser photolysis and stopped-flow is ~10 pM. Yet, steady-state assays of NOD activity reveal much weaker inhibition by NO [52, 135, 341, 344] thus suggesting the existence of mechanisms for decreasing NO binding during catalysis (4)

(4) HbFe2++NO⇌HbFe2+(NO)

The most attractive explanation for this phenomenon is the allosteric modulation of an NO tunnel or gate by O_2_ binding. In the *M. tuberculosis* trHbN, O_2_ and NO access the ferrous heme through two different gated tunnels [345]. O_2_ binding opens a long tunnel for NO and increases NO access [346–348]. Tunnel switching occurs on the picosecond to nanosecond time scale and is compatible with a role in NOD catalysis [349]. A novel role for PheE15 in gating O_2_ and NO migration through channels has been suggested in which the distal H-bonding TyrB10 and GlnE11 act as the triggers [348, 350, 351]. Similar tunnels or gates may control NO access in other Hbs. Bis-histidyl ligation and structural plasticity may serve a similar role in controlling ligand access in the NOD function of plant nsHb [84, 352].

 Other explanations for diminished NO inhibition include NO reduction by the flavoHbs [341], yet, rates of NO reduction by some flavoHbs appear too low [52]. Interestingly, Liu et al. were able to mathematically model the Cygb-NOD activity with the available transient kinetic rate constants for NO and O_2_ binding and NO dioxygenation [136] suggesting the absence of mechanisms for decreasing NO binding in some Hbs.

### 11.3. Superoxide Radical-Like Character of the Bound O_2_

 The ferrous heme in Hb transfers an electron to O_2_ to form a stable Fe^3+^(O_2_^−^) complex [353, 354]

(5) HbFe2+(O2)⇌HbFe3+(O2−)

*The ferric iron forms unique bonding interactions with the bound O*_2_^−^ *important for NOD function*. The heme-Fe^3+^ (3*d* ^5^) unpaired electron interacts with the unpaired π*O_2_^−^ electron through strong antiferromagnetic coupling in a unique end-on orientation [103]. In the NO dioxygenation reaction, the unpaired NO π* electron associated with the N-atom couples with the unpaired π*O_2_^−^ electron. One electron is formally transferred from NO to the half-filled π* (O_2_^−^) orbital (reduction of superoxo), but not to the metal; thus the strong *π* O_2_ bond is broken and the Fe-O bond is strengthened. The reaction is analogous to the diffusion-limited reaction of NO and O_2_^−^ in solution [355] except that *the strong anti-ferromagnetic coupling of an end-on O*_2_^−^ has the capacity to preferentially localize the unpaired π* electron density to the O-atom proximal to the iron and to direct the NO reaction (*vide infra*).

 The anti-ferromagnetic coupling is eliminated with the return of the electron from the bound O_2_^−^ to Fe^3+^ permitting O_2_ dissociation. However, many Hbs that function as NODs (e.g.,* E. coli* flavoHb) show relatively high autooxidation rates and O_2_^−^ release. Toxic O_2_^−^ release is more problematic at high oxy-Hb concentrations and is slow for abundant Hbs and Mbs functioning in O_2_ transport-storage [356–358]. In contrast, the less abundant neuronal Ngb shows high autooxidation rates *in vitro* [359]. Curiously, this has led some investigators to discount a NOD function [360]. However, high autooxidation rates can be an artefact of *in vitro* conditions and should not be the sole basis for conclusions about function. For example, autooxidation of the *Candida norvigensis* flavoHb-NOD oxy-complex is very slow, but increases when the reductase domain cofactor FAD is absent or when the reductase domain is separated from the Hb domain [61, 102].

### 11.4. Protected Pocket for the Peroxynitrite Intermediate

 A transient ferric-peroxynitrite intermediate likely forms in the reaction of NO with globin Fe^3+^(O_2_^−^) [65, 66] (6)

(6) HbFe3+(O2−)+NO→HbFe3+(ONOO−)

The half-life of the fleeting peroxynitrite intermediate is expected to be in the microsecond range due to facile iron-catalyzed isomerization to ferric-nitrato, whereas the ferric-nitrato species has a measured half-life of milliseconds [361, 362]. The putative HbFe^3+^(ONOO^−^) intermediate detected in reactions of oxy-Mb, -Ngb and -GlbO in the millisecond time range [122, 123, 363–365] must now be assigned to the ferric-nitrato species. Oxy-globin model compounds, Fe(Por)(NH_3_)(O_2_), also react with NO at 80–100 K forming only the low-spin ferric-nitrato complexes, thus implying that peroxynitrite intermediates, if formed, also undergo very facile isomerization [366]. In contrast, NO reacts with oxy-coboglobin model compounds, Co(Por)(NH_3_)(O_2_), forming a detectable peroxynitrite intermediate and nitrato species at low temperature [367]. More recently, Navati and Friedman [365] reported preliminary evidence for Fe^3+^(ONOO^−^) formation during the reaction of NO with oxy-Mb and oxy-Hb in a special “dry” glassy matrix.

 The ferric-peroxynitrite would form in the distal pocket of Hb-NODs which is well isolated from nucleophiles and solvent. Hb pockets are typically lined with a large number of hydrophobic residues with histidine, glutamine, and/or tyrosine residues forming hydrogen bonds to the O_2_^−^ ligand. A protected pocket may explain how Hb functions as a high-fidelity catalyst of NO dioxygenation whereas the indoleamine dioxygenase-like oxy Turbo Mb cannot [66].

### 11.5. O-Atom Isomerization or Rearrangement Mechanism

(7) HbFe3+(ONOO−)→HbFe3+(NO3−)

Two mechanisms for formation of nitrate from the reaction of NO and HbFe^3+^(O_2_^−^) (7) have been previously discussed [66]. Both provide a pathway for O–O bond breaking and O-atom isomerization or rearrangement in the peroxynitrite intermediate. The first that we envisioned was an iron-catalyzed mechanism in which ferric iron, acting as a Lewis acid, facilitates O-atom rearrangement [34]. We wrote “The heme-Fe^3+^ may facilitate an oxygen bond rearrangement by participating in a iron-mediated oxygen bond shift analogous to the proton-mediated shift suggested for the nonenzymic mechanism for HOONO decomposition to NO_3_^−^.” The mechanism was never fully rationalized, developed, or argued in the literature, but it was assumed that the mechanism would bear similarity to the mechanism of peroxynitrite isomerization accelerated by acidic pH (pH < 2) [368, 369]. The second, a *ferryl mechanism* involving peroxynitrite, O–O bond homolysis, and ferryl O-atom transfer was strongly supported by theory, overwhelmingly favored, and thoroughly argued and investigated [65, 66, 342, 364]. However, both the doubt cast by recent [121, 362] and earlier [368] investigations and the paucity of strong experimental support for the *ferryl mechanism* have demanded new insights and the scrutiny of alternative O-atom rearrangement mechanisms.

 In 1954, Anbar and Taube [370] suggested a concerted internal O-atom rearrangement mechanism for the isomerization of peroxynitrous acid to nitrate to explain O-atom retention, and a similar mechanism, as already mentioned, was suggested as a possibility for the Hb reaction, albeit remaining poorly defined (see above) [65, 66]. Tsai et al. [371] had argued against an internal rearrangement mechanism for peroxynitrite/peroxynitrous acid in 1996 “because contracting of the O–O–N bond angle produces a strong repulsion between the terminal peroxide oxygen and the two oxygens bound to nitrogen.” Discussions with Henry Taube and recent descriptions of the unique Fe^3+^O_2_^−^ bonding in Hbs [103] provided new insights for a novel concerted internal O-atom rearrangement mechanism (see below). The new mechanism casts doubt on the relevance and validity of mechanistic inferences drawn from experiments demonstrating peroxynitrite isomerization catalyzed by ferric Mb [372–375] or metalloporphyrins [26, 66, 376]. Models of heme-peroxynitrite adducts envision the negatively charged terminal O-atom bonded to iron [376, 377].

**Figure 2:**
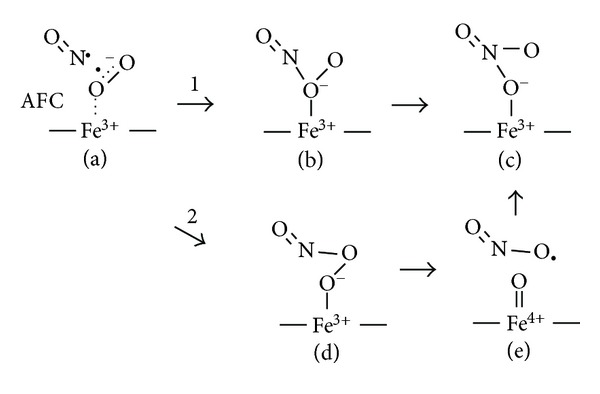
Two possible mechanisms and intermediates for O-atom rearrangement in the NO dioxygenation reaction. (1) shows the *concerted Lewis acid mechanism* in which NO attacks the proximal O-atom and iron facilitates O-O bond heterolysis through electron withdrawal. NO reacts with the antiferromagnetically coupled (*AFC*) ferric-superoxo (a) to form a transient ferric-peroxynitrite intermediate (b), which isomerizes to form the ferric-nitrato species (c). (2) shows the *ferryl mechanism* in which NO attacks the distal O-atom and iron facilitates O–O bond homolysis through electron withdrawal. The NO dioxygenation reaction generates a ferric-peroxynitrite intermediate (d) which weakens the O–O bond to homolysis forming “caged” ferryl and NO_2_ intermediates (e) that then combine to form the ferric-nitrato species (c).

 The two possible O-atom rearrangement mechanisms are illustrated in Figure 2. In the new reaction scheme (1), NO attacks and bonds the O-atom proximal to the iron atom breaking the strong *π* O_2_ bond and strengthening the Fe–O bond, the terminal O-atom attacks the nitrogen, and the O–O peroxide bond heterolytically breaks to form Fe^3+^[NO_3_^−^]. As envisioned, another electron pair donor (e.g., NO, HCN or I^−^) should be able to intercept the terminal O-atom to form NO_2_^−^ and an oxygenated product (e.g., NO_2_, CNO^−^ or IO^−^). *The role of the ferric iron is purely that of a Lewis acid, *as we first imagined [34]*, and the Hb pocket shields reactive intermediates.* Ferric iron forms an ionic or coordinate bond with ONOO^−^, withdraws electrons from the terminal peroxide O-atom, and increases terminal O-atom reactivity with N. In contrast, in the *ferryl mechanism* (2), NO attacks the O-atom distal to the iron atom to form Fe^3+^(ONOO^−^), the O–O bond weakens and homolytically breaks to form the “caged” Fe^4+^ = O (O^−^) and NO_2_ pair which rapidly combine to form Fe^3+^(NO_3_^−^).

 The support for a *ferryl mechanism* was previously reviewed [65, 66, 364] and recently critiqued [121, 362, 375, 378]. From atomistic simulations of the truncated group I HbN, Mishra and Meuwly [121] concluded that the O–O bond scission energy barrier was too high and homolysis too slow thus suggesting an internal rearrangement mechanism. Su and Groves [378] have concluded that NO_2_ is an unavoidable product of the *ferryl mechanism*, yet, there is no evidence for NO_2_ formation or ferryl O-atom scrambling with water during (flavo)Hb-catalyzed NO dioxygenation [66]. One would expect NO_2_-mediated nitration damage to the B10 tyrosine residue in the distal pocket of flavoHb-NOD and a loss of NOD activity similar to the nitrations of more distant tyrosines observed in Mb and Hb [66, 373, 374, 379–383]. The release of reactive toxic intermediates during NO oxidation by oxy-Hb is the antithesis of a NOD function, and the evidence for that is meager [33]. What then are the arguments and evidence suggesting a *concerted Lewis acid mechanism* (Figure 2, (1))? First, the reaction mechanism demands the application of density functional theory and further scrutiny. Past theoretical investigations of peroxynitrous acid isomerization [384] have not considered the effects of hydrogen ion interactions with peroxynitrous acid O-atoms. Nor has O-atom retention during peroxynitrous acid isomerization [370, 385] been investigated as a function of pH. Clearly, hydrogen ions increase the rate of the reaction [368, 369] well beyond the peroxynitrous acid pK*_a_* of 6.5–7 [386], but this phenomenon has never been clarified. A proton, or Lewis acid, interacting with the peroxide O-atom proximal to N would decrease the strong repulsion between the terminal peroxide oxygen and the two oxygens bound to nitrogen. In an analogous reaction, hydrogen ions catalyze O-atom transfer from H_2_O_2_ to I^−^ and other two-electron donors [387–390]. The only apparent catalytic mechanism is through O-atom protonation, which withdraws electrons from the proximal O-atom, increases O or OH^+^ reactivity with I^−^ and which causes peroxide bond heterolysis forming water and IO^−^ or IOH [389]. A Lewis acid (e.g., Fe^3+^) would be expected to be able to catalyze a similar O-atom transfer. There are also reasons why a mechanism involving NO attack of the O-atom proximal to iron in Hbs is attractive. The proximal O-atom is more accessible to NO. Moreover, it is the most probable location for the anti-ferromagnetically coupled unpaired π* Fe^3+^(O_2_^−^) electron [103] that couples with the unpaired NO electron. Hughes and Nicklin had suggested a heterolytic mechanism for peroxynitrous acid isomerization in 1968 [368], but the potential role of hydronium or hydrogen ions in catalyzing the reaction was not considered.

### 11.6. Mechanisms for Nitrate Egress

 Martí et al. simulated nitrate release from *Mycobacterium tuberculosis* trHbN [391]. The molecular dynamic simulations suggest that formation of the ferric-nitrato species causes a structural distortion of the pocket cavity walls forming pores for water entry. Water hydration weakens the bond between the heme iron atom and nitrate exits in ~5 ns *via* a unique pathway differing from O_2_ and NO tunnels. A role for ThrE2 in assisting nitrate egress was proposed. The role of plant nsHb pocket plasticity in nitrate removal has also been discussed [84]. The *Mycobacterium leprae* GlbO ferric-nitrato intermediate showed a 10–100-fold longer half-life than other globins [122] suggesting a slow nitrate egress mechanism.

### 11.7. Mechanisms for Univalent Reduction

 Each NO dioxygenation reaction consumes a single electron that must be supplied by an electron donor for catalytic turnover. The ferric heme is reduced by an electron (8a) for the O_2_ binding reaction (3). Importantly, *Hbs require mechanisms to prevent the transfer of a second electron to the higher potential ferric superoxo complex*. A second electron would generate ferric-peroxide (8b), and a third electron would generate ferryl. Reduction of the putative ferric-peroxynitrite intermediate could release peroxynitrite or generate hydroxyl radical (8c). These reactive species would be expected to cause damage to the heme and/or protein.

(8a) HbFe3++e−→HbFe2+

(8b) HbFe3+(O2−)+e−+H+→HbFe3+(OOH−)

(8c) HbFe3+(ONOO−)+e−+H+  →HbFe3+(ONO−)+OH

Native electron donors are known for only a handful of Hb-NODs, but distinct patterns are emerging. The ultimate electron donors for the flavoHb-NOD [65] and dual function raphidophyte trHb-nitrate reductase [139] are flavin-containing reductases linked by a multidomain structure. The C-terminal flavoHb reductase domain [39, 49] and C-terminal trHb-nitrate reductase domain structures belong to the FNR superfamily which includes NAD[P]H:ferredoxin oxidoreductase and NADH:cytochrome b_5_ oxidoreductase. The truncated cyanoglobin (GlbN) and ferredoxin reductase genes are also linked in a bi-cistronic operon controlled by the NO sensor-regulator NsrR in *Legionella pneumophila* [239]. Together, the data suggest a common role for ferredoxin reductases and other FNR-like proteins as electron donors for trHbs and SDGs either linked in multi-domain structures or as separate proteins. The role of the of pre-A sequence for truncated HbN-NOD activity in Mycobacteria may be to facilitate interactions with a specific ferredoxin reductase [392]. Ferredoxin:NADP^+^ oxidoreductase from *E. coli* was systematically tested for its capacity to support NOD activity of various Hbs *in vitro* [133], however, in those reactions, “NOD activity” is impossible to discern from reactions of O_2_^−^. High concentrations of SOD are required to prevent reactions of NO with the O_2_^−^ invariably released by reductases [344]. Red blood cell Hb is reduced by cytochrome b_5_, and cytochrome b_5_ can support the Cygb-NOD activity *in vitro* [135]. Ascorbate also supports the Cygb-NOD activity at concentrations found within Cygb expressing fibroblasts and neurons [135, 136], and a putative binding site for ascorbate has been identified [135]. An accessory role for NADPH:cytochrome P450 oxidoreductase in CygbFe^3+^ reduction has also been suggested [136]. The barley nsHb can be reduced by an ascorbate-dependent monodehydroascorbate reductase [132]. A mass spectrometry approach has been utilized to identify the Ngb and Mb interactome [393]. The method may also reveal novel electron donor candidates.

**Figure 3:**
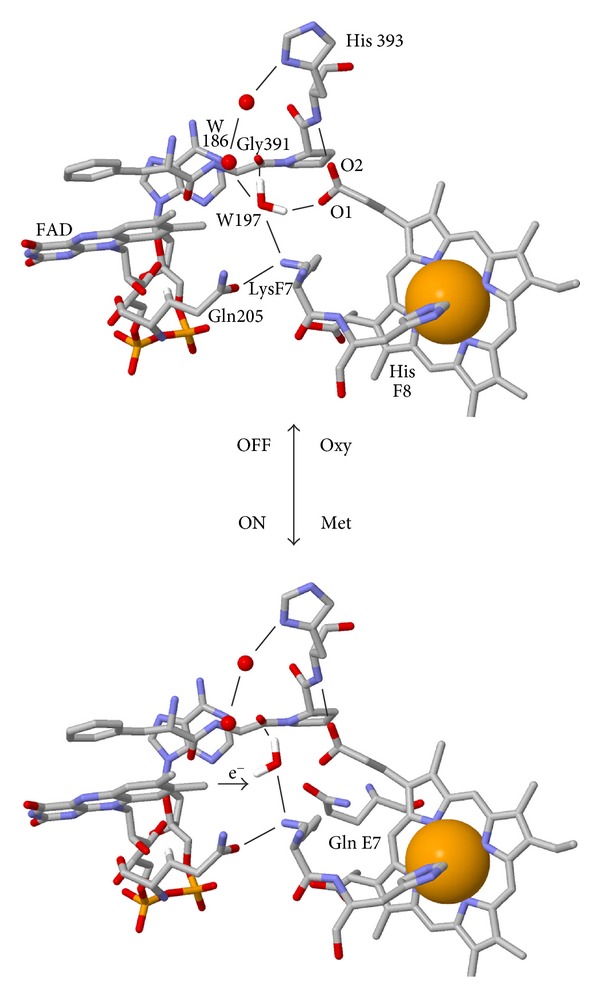
Proposed rotation of a bridging water molecule as a mechanism for electron transfer control. *E. coli* flavoHb electron transfer bridge structure showing proposed changes in the orientation of the bridging water molecule (W197) leading to water polarity changes and ON and OFF states controlling electron transfer.

 Hb structures suggest two mechanisms for controlling univalent electron transfer. Bis-histidyl ligation in hexa-coordinate nsHbs, Cygb, and Ngb provides structural plasticity [84] and a mechanism for controlling electron donor binding and electron transfer. For example, movements of the E-helix and ArgE10 with changes in O_2_ binding may modulate ascorbate binding and electron transfer in Cygb [135]. In this case, bis-histidyl ligation to the ferric iron would be expected to *induce an ascorbate binding-site*, and bis-histidyl ligation to Fe^3+^(O_2_^−^) to decrease ascorbate binding. *A rotating water bridge may also provide a mechanism for controlling univalent electron transfer from reduced flavins.* The flavoHb and SDG structures [49, 65] and electron pathway analyses [394] suggest an important role for water molecules bridging the FAD and heme in mediating electron transfer. In the *E. coli* flavoHb-NOD structure [339], the bridging water molecule hydrogen-bonded and anchored by LysF7 ammonium group has the capacity to rotate to ON and OFF orientations (Figure 3). Strengthening of a short, strong hydrogen bond between water and the heme propionate, with O_2_ binding and a pK*_a_* shift of the heme propionate, provides a mechanism for controlling the bridging water orientation and regulating univalent electron transfer in Hb-NODs. The oxygen core repels electrons in the OFF orientation and would block electron tunneling. In support of the hypothesis, mutation of LysF7 dysregulates univalent electron transfer, oxidatively destroys the heme, and incapacitates the NOD function [395].

 The many adaptations of the Hbs for the NOD function show that Mother Nature is indeed a brilliant chemist!

## 12. The FlavoHb Denitrosylase (O_2_ Nitrosylase) Concept

 Following the report of NO metabolism by the NO-inducible *E. coli* flavoHb and kinetic investigations supporting a NOD mechanism [34, 52, 341], Hausladen et al. argued for a major revision by suggesting a denitrosylase mechanism for NO conversion to nitrate [396, 397]. NO binding to the ferrous heme, and Fe^2+^ reducing NO to Fe^3+^(NO^−^) and N_2_O was reported, and evidence for a reaction of O_2_ with Fe^3+^(  NO^−^) to form nitrate (9) under more relevant physiological O_2_ and NO concentrations was argued. The rationale for the denitrosylase hypothesis was largely motivated by transient kinetic measurements showing a 1500-fold greater affinity of the ferrous heme for NO than O_2_ [52, 341] coupled with the observation of flavoHb-catalyzed NO metabolic activity at an exceptionally high ~1 : 6 NO : O_2_ ratio [398]. Hausladen et al. concluded that the NO and O_2_ affinities necessarily indicated an unfeasible competition of O_2_ for the flavoHb heme under physiologically relevant conditions. Thus, the high NO affinity supposedly negated any possibility of a NOD mechanism at physiological O_2_ and NO levels. The argument epitomized the dogmatic belief that NO binding to any ferrous hemoglobin would prevent O_2_ binding and thus a catalytic NO metabolic activity that was dependent upon O_2_ binding. NO competition, or the lack thereof, remains relevant in investigations and assignments of a NOD function-mechanism for various hemoglobins (see above) [133, 135, 136, 344], but it does not prohibit the mechanism

(9) HbFe3+(NO−)+O2→HbFe3++NO3−

Despite a dearth of either experimental or theoretical support for the flavoHb denitrosylase mechanism after more than 10 years, a handful of investigators persistently assign merit to the mechanism [67, 69, 70, 215, 250, 399] or equate the two mechanisms [87, 400] and in so doing obscure the true function of (flavo)Hbs and impede general progress. Moreover, proponents of the denitrosylase mechanism continue to reject the dioxygenase mechanism and nomenclature, but have received only a partial scientific rebuttal [65, 344, 397, 401]. Hence, a refutation of the denitrosylase mechanism is demanded.

 The following is a list of reasons why a denitrosylase mechanism is theoretically unfeasible and weakly supported by experiment. (1) NO reduction (*E* ^0^ = −0.8 V)[189, 402] to the nitroxyl (NO^−^) intermediate has a large energy barrier. *V*_max⁡_ values for NO reduction by flavoHb are >2000–fold lower than the *V*_max⁡_ values for NO dioxygenation [52]; (2) the reaction of Fe^3+^(NO^−^) with O_2_ is not kinetically favored [123, 403]; (3) O_2_ would be more rate-limiting for a denitrosylation at low physiological O_2_ concentrations than it would for the NOD mechanism; (4) the mechanism requires O_2_ to react with the N-atom liganded to the iron which does not provide an obvious reaction path for O-atom rearrangement; (5) O_2_ binding and NO scavenging are affected by the flavoHb distal pocket structure (TyrB10), but NO binding is not [341]; (6) CO competitively inhibits NO metabolism with respect to O_2_, not NO, throughout the physiological [O_2_] range [52, 344]; (7) the NO metabolic activity of flavoHb is saturable by O_2_ [52, 341, 344] with no evidence of an activity increase at low O_2_ as suggested and reported [396, 398]; (8) NO inhibits the NO metabolic activity of flavoHb at NO:O_2_ ratios >1 : 100 and not as potently as predicted from NO affinity measurements [52, 341, 344]; (9) flavoHb-NOD activity is induced by NO providing an effective escape from the otherwise problematic NO inhibition at low [O_2_] [151, 404]; (10) NO reductases are also expressed at low O_2_ concentrations thus substituting for lost flavoHb function due to NO inhibition [151, 404]; (11) the denitrosylase proposal assumes that micromolar NO concentrations are required for NO toxicity and thus the NO detoxification function of flavoHb. Presumably, under these high NO levels, a dioxygenase mechanism would be impossible. But, even a “tiny” 50 nM NO is toxic to aerobic *E. coli* [35] and flavoHb protects against this toxicity [35, 151, 404]. Thus, flavoHb-NOD can detoxify NO without significant NO inhibition even at 5 *μ*M O_2_. Furthermore, NOD turnover under normoxia (~400 NO s^−1^ heme^−1^) far exceeds the activity expected from the denitrosylase mechanism. NO reduction by flavoHb, a step in the denitrosylase mechanism, has never exceeded 0.2 NO s^−1^ heme^−1^ [65, 107]. For the two mechanisms to be comparable in rate, the NOD mechanism would need to be inhibited by 99.95%. At 50 nM NO, the available [O_2_] would need to be <5 nM for 99.95% NOD inhibition for a remotely plausible and equivalent NO scavenging function. (12) Finally, the initially troubling observation of flavoHb metabolizing NO and NADH at an NO : O_2_ ratio of >1 : 6 [398] can be explained by the fact that unusually high levels of flavoHb (0.5–1 *μ*M) were present in the reactions such that the added NO (35 *μ*M) was so rapidly consumed as not ever to achieve >5 *μ*M NO! (see Figure  1(a), [398]). Moreover, the substantial NADH oxidase activity of flavoHb can account for NADH oxidation observed at these high concentrations of flavoHb (see Figure  1(d), [398]). These unfavorable conditions for NOD catalysis may also explain the relatively high yield of nitrite formation and would even compromise the deduced O_2_ : NO : NADH reaction stoichiometry [398]. The concentrations of flavoHb protein used in steady-state kinetic analyses are typically 5,000-fold lower and involve high turnover numbers [52, 341, 344].

*Unresolved issues nevertheless remain. For example, why is the affinity of flavoHb for NO ~15-fold greater when determined by the flash photolysis-rebinding method than when estimated from steady-state kinetic analysis*? NO removal by the flavoHb NO reductase activity during catalysis is one possibility [341], but the activity may be insufficient to account for NO resistance. Similar questions arise for all other globins including the mammalian Cygb. The Cygb NO : O_2_ affinity ratio of ~125,000 : 1 immediately suggests a much greater inhibition by NO than that observed at NO : O_2_ ratios of >1 : 500 [135]. Liu et al. [136] have recently used modeling simulations to argue that the steady-state kinetics measured for the Cygb-NOD activity are allowed by the high NO affinity (*K*_*D*_ = 8 pM), but the model required assumptions including NO affinity decreases with temperature. Less consideration has been given to structural dynamics and changes in NO access [346, 348] as a common mechanism for limiting NO binding and inhibition of catalysis (*see above*).

## 13. Why So Many Different Hb-Type NODs?

 While there is certain danger of a fallacy of composition in suggesting a NOD function for all Hbs, the literature now shows that many different Hb structures in many different forms of life function as NODs. This realization raises important questions. If various Hbs function as NODs, why the structural diversity and the multiplicity of Hbs within organisms?

 Cursory answers to these questions can be readily gleaned from the anti-oxidant and radical scavenging enzyme systems. For example, in order to scavenge H_2_O_2_, organisms express catalase, glutathione peroxidases (8 isoforms in humans), cytochrome c peroxidase, ascorbate peroxidase, and peroxiredoxins (6 isoforms in mammals) [12]. And, for O_2_^−^ removal, multiple SOD isoforms are expressed (7 isoforms in Arabidopsis) [11, 15, 405]. These *enzyme isoforms show differential localization in subcellular compartments, expression during development and stresses, electron sources, catalytic requirements, posttranslational regulation, and subtle differences in physiological functions.*

 The analogy implies a similar ubiquity and richness of NO- and Hb-related functions in nature. Moreover, examples are emerging to support this rubric. *Aspergillus* flavoHb-NODs are differentially distributed to the cytosol and mitochondria because FHb2 bears a mitochondrial N-terminal signal sequence [112, 216]. A class I truncated Hb localizes along chloroplasts thylakoid membranes in algae [406]. Other examples of Hbs with membrane localization signals or affinities include the *M. tuberculosis* HbO [407, 408], VHb [409, 410], the myristoylated Crab globin [411], and the myristoylated or palmitoylated fish GlbX [88]. Membrane association may also facilitate reduction by membrane-bound reductases. *Saccharomyces cerevisiae* flavoHb localizes to mitochondria and cytosol without an apparent signal sequence [412]. The wheat nsHb interacts with, and apparently cofunctions with, photosystem I and II [331]. Many Hbs show tissue-specific and developmental regulation of expression [82, 116, 222, 296, 304, 336, 413]. Some Hb-NODs are induced by NO stress, and others are not [95, 116, 216, 310]. Some flavoHb-NODs prefer NADH while others prefer NADPH [52, 114, 216], and expression appears to correlate with the availability of the electron source. The Cygb-NOD utilizes ascorbate as an electron donor *in vitro* [135, 136] and is expressed in ascorbate-rich fibroblasts and neurons [296]. Transcript network analysis strongly suggests a role for ferredoxin: NADP^+^ oxidoreductase as the electron donor for cyanoglobin (GlbN) in *Legionella pneumophila* [239], and GlbN would be required to interact specifically with the reductase. Some globins are suited for NOD catalysis at relatively high micromolar NO concentrations and fluxes (e.g., flavoHbs) [52, 341] whereas others show lower turnover rates and are more susceptible to NO inhibition (e.g., Cygb and Ngb) [135]. This is reasonable since some globins serve primary NO detoxification functions while others subserve NO signaling functions. Some Hbs may function better at low or high temperatures [111, 219] while others may function under dehydrating osmotic stresses [308]. Potential mechanisms for post-translational regulation are also emerging. Reeder et al. have reported that lipid binding to Cygb alters heme coordination and suggest that this provides a mechanism for lipids to alter or regulate Cygb function [414]. Protein phosphorylation and thiol-disulfide interchange have been suggested as mechanisms regulating Ngb function [415–417]. In addition, some globins (e.g., muscle Mb and legume Hb) may be suited to serve dual functions in O_2_ transport-storage and NO metabolism [125, 137, 255, 262]. *These many differences are achieved by many unique Hb structures while intrinsic O*_2_ *binding and NO dioxygenation activity appear to be preserved in all Hbs.*

## 14. Are Hemoglobins Unique O_2_-Dependent Catalysts for NO Metabolism?

 The simple rapid bimolecular reaction between NO and O_2_^−^ to generate peroxynitrite (*k*_2_ = 6.9 × 10^9^ M^−1^ s^−1^) [189, 355], and ultimately yield ~70% nitrate, initially suggested only minimal requirements for efficient O_2_-dependent NO metabolism in biological systems [284]. While attractive, the very serious consequences of peroxynitrite toxicity [189, 190] were often overlooked. It is now clear that many organisms metabolize NO to nitrate *via* Hb-type NODs. What is not clear is whether Hb is the only protein family capable of catalyzing significant O_2_-dependent NO decomposition in cells. Other O_2_-binding heme or copper catalysts yield peroxynitrite reaction products [26, 418] or scramble the O-atoms with water [66] suggesting a unique capacity of Hbs for high-fidelity NO dioxygenation. On the other hand, an oxygenated *Rhodobacter spaeroides* cytochrome apparently dioxygenates NO *in vitro*, forms a complex with an electron-carrying b-type cytochrome and may function as a novel NOD [419].

 In many cases, the catalysts of O_2_-dependent NO metabolism in microbes, plants, brain tissue [199, 271, 274, 420, 421], lung tissue [422], liver parenchymal cells [275], various cultured cell lines [142, 423], endothelial cells [424], macrophages [425], and the aortic wall [426, 427] remain to be defined. Besides the Hbs, cytochrome c oxidase [428], succinate-cytochrome c reductase [429], dihydrolipoamide dehydrogenase [430], and cytochrome P450s [423] join a growing list of cellular NO removal catalysts put forward for consideration.

## 15. Other Enzymatic Functions for Hemoglobins?

 In addition to NO dioxygenation, several specialized enzymatic activities and functions have been proposed for various Hbs. Discerning a meaningful biological function from myriad fortuitous *in vitro* activities of Hbs [65] continues to be an important challenge. In all cases, investigators must always ask whether (1) other enzymes coexist in cells that could better serve the proposed function, (2) the structure evolved for the proposed function, and (3) the reaction is beneficial for long-term survival of an organism. In evaluating proposed functions of Hbs, it is also important to consider the fundamental argument of Shikama and coworkers [61, 102] that (4) *“the reversible binding of molecular oxygen to iron*(II) *must be the primary event to manifest their physiological functions in vivo.”* Of the following recently proposed enzymatic functions, surprisingly, only the heme oxygenase activity meets the last and most basic of these criteria.

### 15.1. NO Reductase

 Anaerobic NO reduction was first suggested to be an important biological function for the *E. coli* flavoHb by Kim et al. [107]. The reaction (10) served to explain the anaerobic induction of flavoHb by NO*_x_* [50] and the anaerobic growth protection flavoHb provided against nitrosothiols [55].

(10) 2 NO+2  e−+2 H+→N2O+H2O

However, the proposed function has critical weaknesses. For example, other microbial flavoHbs show much lower [52], or negligible [48], NO reductase activity. The maximal turnover rate for NO reduction is >2000-fold lower than that measured for NOD activity, and is unstable (*unpublished results*). Furthermore, the flavoHb shows no NO reductase activity within anaerobic *E. coli* [404]. Moreover, there are more efficient NO reductases in organisms expressing flavoHb or other Hbs [17, 151], and flavoHbs are capable of reducing nitrosothiols directly [52], thus explaining the protection observed. Yet, uncertainty, and confusion, over a NO reductase function clearly persists in the field of nitrosative and oxidative stress research [431].

### 15.2. Peroxynitrite Isomerase

 The commonly accepted NOD mechanism includes the efficient isomerization of a ferric heme-bound peroxynitrite intermediate to nitrate (7). Given the formation of toxic peroxynitrite in cells from the rapid reaction of NO and O_2_^−^, Herold and others have suggested that various Hbs and Mb may also function as scavengers of peroxynitrite [125, 138, 432]. However, large rate constants (>10^6^ s^−1^ M^−1^) for peroxynitrite isomerization were only measured for distal E7 histidine mutants [372] suggesting that entry, binding or isomerization of the ONOO^−^ anion in the heme pocket is normally hindered by E7 histidine. Exposure of Mb or Hb to exogenous peroxynitrite also nitrates and damages the protein [373, 374, 379–383]. Hence, these Hb structures do not appear adapted for a peroxynitrite isomerase function. Secondly, peroxynitrite reacts with a variety of abundant biomolecules (e.g., CO_2_ and glutathione) [189], and these biomolecules would compete with ferric Hb or Mb. Moreover, proposals of a peroxynitrite isomerase function ignore (1) the important function of SODs and NODs in preventing toxic peroxynitrite formation within cells and (2) reactions with the predominantly oxy Hb within cells [382, 383].

### 15.3. Nitrite Reductase

 As early as 2003, Mark Gladwin and others began pursuing possible NO-generating nitrite reductase functions for the mammalian red blood cell Hb [433, 434] and muscle Mb [269, 435, 436]. The reaction (11) produces NO from nitrite under aerobic and hypoxic conditions *in vitro* and *in vivo* and is stimulated by acidic conditions that occur in tissue ischaemia

(11) NO2−+e−+2H+→NO+H2O

A vital role for the reaction in the hypoxic vasodilation of capillaries and homeostatic control of tissue O_2_ delivery was proposed. In addition, the reputed nitrite reductase function has been extended to other members of the Hb superfamily. For example, dithionite-reduced Ngb catalyzes NO production from millimolar nitrite under anaerobic conditions with rate constants estimated at 0.12 to 5 s^−1^ M^−1^ [416, 437]. Furthermore, Gladwin and co-workers have reported effects of distal histidine interactions, disulfide bond formation, and protein phosphorylation on the rate of the deoxy Ngb-nitrite reaction [415, 416]. The reaction rate constant increased to physiologically relevant values (~50 s^−1^ M^−1^) with the elimination or weakening of the distal histidine-heme interaction. Li et al. reported similar low levels of nitrite reducing activity (0.14 s^−1^ M^−1^) for Cygb and demonstrated a significant role for the activity in soluble guanylate cyclase activation in cultured aortic smooth muscle cells with 10 *μ*M nitrite [438]. This is remarkable since the theoretical maximum rate of NO generation under these conditions with an upper [deoxy-Cygb] estimate of 1 *μ*M is 1.4 pM s^−1^. Neighboring endothelial cells normally generate and release NO at a far higher rate of 17 to 1500 nM s^−1^ [277, 278]. As pointed out by Sturms et al., “the modest (low micromolar) levels of nitrite typically found in mammalian tissues diminish the likelihood that nitrite reductase activity is a major function of these proteins” [439]. Rather, Sturms and Hargrove suggest that plant hexacoordinate Hbs may be more suited to nitrite reduction since nitrite levels are higher in plant tissues. They have demonstrated anaerobic nitrite reduction by deoxy forms of rice Hb and *Synechocystis* Hb with respective bimolecular rate constants of 166 and 130 s^−1^ M^−1^ [439], but these Hbs are of relatively low abundance. Tiso et al. subsequently reported comparable bimolecular rate constants of 5 and 20 s^−1^ M^−1^ for the *Arabidopsis thaliana* nonsymbiotic Hbs [440].

 It is important to note, however, that the reaction of nitrite with globins nitrosylates ferrous heme and cysteine sulfhydryls [437] and that the reaction of nitrite with globins in the presence of H_2_O_2_ nitrates proteins [441, 442]. Moreover, high nitrite concentrations under mildly acidic aerobic conditions form nitriheme, a damaged heme [443]. Thus, the anoxic NO_2_^−^ reduction reaction is likely a non-specific and damaging reaction of Hbs. Furthermore, the NO-generating reactions of Hbs must always be evaluated with other enzyme systems that also act as non-specific nitrite reductases [211] including the plant nitrate reductase [204] and mitochondria [444].

### 15.4. Hydroxylamine Reductase

 Sturms et al. have suggested an additional role for plant and cyanobacterial deoxyHbs in reducing hydroxylamine (12), an intermediate in nitrite reduction, under anaerobic conditions [445]. The proposed role presumes a failure of the nitrite reductase to adequately fulfill the function

(12) NH2OH+2 e−+2 H+→NH3+H2O  

### 15.5. Alkylhydroperoxide Reductase

 Bonamore et al. have reported an anaerobic alkyl hydroperoxide reductase activity for *E. coli* flavoHb with turnover rates of ~1 s^−1^ [71, 446]. Given the high affinity of the flavoHb distal pocket for hydrophobic lipids, the authors have suggested a function for flavoHbs and related single domain Hbs in protecting cells from lipid-related oxidative stress. However, the benefit of flavoHb for protecting cells against peroxide stress is questionable given the exquisite sensitivity of the heme to destruction by peroxide(s) [447].

### 15.6. Peroxidase

 Numerous peroxidative activities of Hbs have been demonstrated *in vitro* suggesting potential peroxidase functions. Recent suggestions and investigations of Hbs functioning as peroxidases include the *Synechocccus* GlbN (a class 1 trHb) [138], Cygb [448–451], Ngb [450, 452], and several Arabidopsis Hbs [441]. As pointed out by Paul [453], when evaluating a peroxidase function, one needs to determine overall turnover rate, rates of elementary steps, and specificity for electron donors and compare them with those reported for genuine peroxidases. A peroxidase function must also be demonstrated within the living organism in the normal background of peroxidases and catalase. Only in the case of the peroxidative dehalogenation of phenols by the polychaete *Amphitite ornate* Hb [454] has a peroxidase function been convincingly demonstrated.

### 15.7. Lactate Dehydrogenase

 Gupta et al. [118] have recently reported the failure of *Mycobacterium tuberculosis* flavoHb [Rv0385] to meet the qualifications of a NOD. The protein is hexa-coordinate and shows very low NOD activity [119] and little ability to protect against nitrosative stress imposed by acidified nitrite when expressed in *E. coli* or *M. smegmatis* [118]. A function as a D-lactate specific dehydrogenase was suggested. The proposed mechanism is similar to that of the L-lactate oxidizing flavocytochrome b_2_ [72] where lactate transfers electrons to the flavin, and the flavin transfers electrons to the heme. A rather low *in vitro* turnover number for D-lactate oxidation of 0.026 s^−1^ is estimated from the published data [118]. The possibility of post-translational modifications influencing activity was not considered. Interestingly, the putative *M. tuberculosis* flavoHb (Rv3571) was previously suggested to function in NO detoxification [455], but the work has not been confirmed or extended.

### 15.8. Electron Carrier

 Other electron transfer functions, absent a role for O_2_, have been suggested for Hbs. Brittain et al. have suggested that the hexa-coordinate Ngb transfers electrons to the mitochondrial cytochrome c to prevent apoptosis [108], and additional roles in reductive stabilization of Hif-1*α* and Nrf2 during hypoxia have recently been argued [456]. However, Ngb-deficient mouse models do not support a role for Ngb in preventing apoptosis during hypoxia [303]. The reported changes in the Hif-1*α*-regulated transcription response in Ngb-deficiency [303] may be explained by impaired NO metabolism and NO inhibition of the Hif-1*α* destabilizing prolyl 4-hydroxylase [170, 171].

### 15.9. Heme Oxygenase

 The flavoHb heme is readily destroyed and iron is released upon exposure to H_2_O_2_ [447]. FlavoHbs, and presumably other Hbs, thus have the capacity to act as heme oxygenases. Moreover, multielectron reduction of the heme-bound O_2_ to H_2_O_2_ and/or ferryl by the reductase domain of a flavoHb (8b) generates the necessary intermediates for initiating the heme oxygenase-like mechanism [457]. When the distal TyrB10 and His E7 residues are mutated in the *C. jejuni* truncated Ctb, Ctb can act as a heme oxygenase [226].

### 15.10. Anaerobic CO Metabolism

 The 3.8–4.1-billion-year-old *Methanosarcina acetivorans* protoglobin [MaPgb] binds CO tightly and is thought to function in anaerobic CO fixation possibly through CO interactions [110]. An O_2_-dependent NOD function appears to have been discounted because the organism is “strictly” anaerobic. However, the authors ignore the fact that many strict anaerobes tolerate brief O_2_ exposures in their natural habitats. Indeed, the genome of *Methanosarcina acetivorans *contains genes for the O_2_ defensive enzymes Cu,Zn-SOD (*sodC*) and catalase/peroxidase (*katG*). Moreover, assumptions of an origin of O_2_-generating reactions with increasing atmospheric O_2_ concentrations (~2.45 billion years ago) ignore uncertain O_2_ utilization rates and potential pockets of O_2_ generation on the Archaean Earth [458].

## 16. DNA-Binding and Other Regulatory Functions of Hb Domains

 There are numerous examples of the Hb scaffold being used by Nature as part of multi-domain DNA-binding transcription regulators and enzymes including kinases, guanylate cyclases, and phosphodiesterases [1]. These ‘globin-coupled sensors' were outlined and discussed by Maqsudul Alam and his colleagues in their 2005 review [459]. Not surprisingly, the Hb domains in these proteins are generally thought to bind and sense O_2_. They regulate important physiologic functions such as biofilm formation by *Salmonella* [460]. Several of the >33 *C. elegans* globins are fused within multi-domain proteins and likely possess O_2_-sensing regulatory functions [2, 3]. Whether SDGs function in a similar capacity, but in non-covalent association with enzymes, is an important consideration when evaluating the function of Hbs. For example, oxidized ferric Ngb has been found act as a heterotrimeric G*α*-protein guanine nucleotide dissociation inhibitor and has been proposed to regulate G*α*-protein signaling [461, 462].

 Importantly, the facile NO dioxygenation reaction may also be relevant to the biological function of some multi-domain sensor-regulators. For example, the O_2_ binding *Azotobacter vinelandii Av*GReg, a globin-coupled sensor, reacts rapidly with NO and is thought to serve a NO detoxification [463] or NO sensing function. In the case of the *Mycobacterium tuberculosis* PAS domain sensors DevS and DosT, NO rapidly reacts with the oxy forms to generate nitrate and the ferric heme form [196]. The putative NO dioxygenation reaction triggers the dormancy program through subsequent formation of the kinase activating ferrous-NO form. The results are intriguing and may explain the ability of NO to induce and maintain the antibiotic-resistant dormant state of the bacterium in tuberculosis [464, 465]. A similar NO dioxygenation reaction of the FixL regulator may explain a major part of the NO transcriptional response of *Sinorhizobium meliloti* [183].

## 17. Hb-NOD Technologies

 Progress in understanding the NO-scavenging NOD function and mechanism of various (flavo)Hbs continues to inspire medical, agricultural, and industrial inventions and is helping to unravel the complex biology of NO in a variety of organisms.

 For example, human red blood cell Hb has been modified and is being tested for use as an injectable long-lived NO and oxidant scavenger for treatment of septic shock [466]. Future designs that incorporate a catalytic Hb-NOD activity are anticipated. Alternatively, small molecule NOD mimetics [26] may find therapeutic applications once shielding of reactive intermediates in the NO dioxygenation reaction can be achieved.

 Mechanistic inhibitors of NODs are being investigated for therapeutic use against microbial infections, hypertension, and malignant tumors [467–469]. Imidazoles bearing bulky hydrophobic groups such as the antifungal agent miconazole are particularly effective inhibitors of the flavoHbs [469]. X-ray crystal structures of miconazole and other imidazoles with *R. eutropha* flavoHb reveal key interactions with hydrophobic residues (e.g., Ile25) in the distal heme pocket [470]. These interactions suggest strategies for the rational design of therapeutic inhibitors such as the addition of specific hydrophobic groups to the miconazole nucleus. Imidazoles may also be especially effective towards *S. aureus* and other microbes. Miconazole increases O_2_^−^/H_2_O_2_ generation by flavoHb in *S. aureus* in addition to blocking the NOD function [471].

 In agriculture, Monsanto Corp. is engineering corn, soybean, and other crop plants expressing *E. coli*, *Erwinia chrysanthemi* and yeast flavoHb-NODs for increased resistance to stresses generating NO, increased capacity for soil nitrogen (NO) sequestration in the form of nitrate, and improved growth and fruit production characteristics [472]. Using the patented technology, projected growth and fruit yields increase by ~20% and ~5%, respectively. Many others have engineered transgenic plants to express the *Vitreoscilla* Hb, or other SDGs with the capacity for catalytic NO dioxygenation [75, 129, 256]. The potential beneficial effects for growth, resistance to hypoxia, nitrosative stress, and so forth as well as potential detrimental effects have been recently reviewed [75]. In aquaculture, hypoxia resistant fish are being created through uniform expression of the *Vitreoscilla* Hb directed by the carp actin promoter [257].

*Vitreoscilla* Hb is now being frequently exploited to increase the industrial production of proteins and other molecules by bacteria and yeasts under hypoxic reactor conditions [473–481], and the primary benefit appears to be increased microaerobic respiration *vis-à-vis* the NOD function [256, 258]. Another potential application is the use of the recently discovered dual function algal raphidophyte NOD-nitrate reductase [139] in bioremediation reactors to scavenge NO produced in fossil fuel combustion and to supply algae with nitrate for increased production of biofuels [482].

 More recently, the flavoHb-NOD has been touted as a molecular tool for determining the role of NO in biological processes [483]. As stressed by Forrester et al. [483], the strategy has clear advantages over pharmacological methods employing NOS inhibitors or gene knock-outs. Indeed, flavoHb and SDGs have already been used to discover and dissect the role of NO in glioma growth and cancer [297], fungal development and mycotoxin production [305], the plant hypersensitive response [115, 224, 484], symbiosis and nodule development [87, 260], N_2_ fixation [183, 184], and plant senescence [485].

## 18. Summary and Outlook

 In all fields, we find change and permanence in thinking [20] and a maturation of obscure phenomena to fundamental theories and advances through the scientific process, as lucidly stated by Werner Heisenberg (1901–1976).

*“For an understanding of the phenomena, the first condition is the introduction of adequate concepts; only with the help of the correct concepts can we really know what has been observed. When we enter a new field, very often new concepts are needed, and these new concepts usually come up in a rather unclear and undeveloped form. Later they are modified, sometimes they are almost completely abandoned and are replaced by better concepts which then, finally are clear and well-defined.”*

Only through the introduction of “*correct concepts*”, controlled experiments, exacting scrutiny, careful retrospection, and the revision or refutation of faulty concepts can basic truths be established from obscure phenomena. This has been aptly illustrated by our progress in understanding the biology of Hb and NO. Sir Humphry Davy's investigations of the “respiration” of *Stickstoffoxyd* (NO/NO_2_) by blood and tissues in 1800 produced the first relevant, yet unclear, phenomena including the remarkable oxidation of the red pigment of blood by the gas [30]. Since then, the red-pigmented proteins Hb/Mb have been shown to carry O_2_ in multicellular organisms, while the NO-metabolizing activity of Hb is more ancient, widespread and only beginning to become clear. An understanding of O_2_ binding remains important for both functions, but *the discovery of the NO dioxygenase function has introduced numerous new questions including some that challenge dogma and traditional views of Hb structure-function. *In addition to continuing to ask which Hbs function as NODs, greater knowledge of enzyme mechanism and biological function are needed to understand the diverse Hb structures and their evolution. A better understanding of the structures governing the confounding competitive binding of O_2_ and NO to the ferrous heme during catalysis, or rather lack thereof, is requisite. Catalysis also needs to be understood in terms of the relevant *in vivo* concentrations of NO and O_2_. Also, much remains to be discovered in the area of electron donors and electron transfer.

 Knowledge of the NOD activity and function of various Hbs and flavoHbs is being applied to the development of NOD inhibitors for use as therapeutic antibiotics, antitumor agents, and vasomodulators. FlavoHbs are being widely used as tools to assess the involvement of NO in the physiology and pathophysiology of plants, animals, fungi, and bacteria. Plants are being genetically modified for (flavo)Hb expression for increased NO resistance, nitrogen assimilation, growth, and crop yields. Hbs and flavoHbs are also being used to increase the productivity of bacteria, yeasts, and algae in biotechnological and environmental remediation applications. It is hoped that success in any of these or other applications provides tangible rewards for past and current efforts aimed at understanding the NOD function of Hbs.

 Finally, I end with a tribute to the spirit of the discoverer of the “Sauerstoffsaugung” (Hb's O_2_-absorbing function) and pioneer of molecular biology, Friedrich Hünefeld [486], by echoing his motto 172 years later.

*Whatever is still hidden reveals ages in the light!*

## Figures and Tables

**Table 1:** Some important biological targets and actions of NO.

Target Reaction	Sensitivity (Est.)	Consequence	Reference
Aconitase (mitochondrial)	>50 nM	Citric acid cycle inhibition	[141–147]
IRE-BP (cytosolic aconitase)	Nanomolar	Iron homeostasis	[143, 146, 148–150]
6-phosphogluconate dehydratase	>50 nM	Entner-Doudoroff pathway inhibition	[151]
Dihydroxy acid dehydratase	Nanomolar	Branched chain amino acid deficiency	[152–154]
Iron-sulfur enzymes (e.g., dehydratases above)	Nanomolar	Formation of toxic iron-dinitrosyl complexes	[146, 155–158]
Cytochrome oxidase (and other terminal oxidases)	Nanomolar	Respiratory inhibition	[141, 142, 159–166]
Catalase	Nanomolar	H_2_O_2_ damage	[167–169]
Prolyl hydroxylase family	Nanomolar	Hif-1*α* stabilization and hypoxic response, collagen cross-linking	[170, 171]
Cytochrome P450 family	Nanomolar	Metabolism of hormones, lipid second messengers, and so forth, Heme release and damage.	[172–174]
Ribonucleotide reductase (diiron)	Nanomolar-micromolar	Inhibition of DNA synthesis	[175–178]
Heme oxygenase family	Nanomolar	Inhibition of toxic heme breakdown	[179, 180]
Photosystem II	?	Inhibition of photosynthesis	[181]
Nitrogenase	Nanomolar-micromolar	Inhibition of N_2_ fixation	[182–187]
Hydrogenase	Nanomolar	Inhibition of N_2_ fixation	[188]
O_2_	Micromolar	NO_2_ damage	[189]
O_2_^−^	Nanomolar	Peroxynitrite damage	[189, 190]
Guanylate cyclase	0.1–10 nanomolar	cGMP kinase activation and smooth muscle relaxation	[191, 192]
Transcription regulators (NorR, NsrR, DevS, etc.)	Nanomolar	NO defense gene expression	[80, 193–196]
ACO (1-aminoacyl cyclopropane-1-carboxylic acid oxidase)	Nanomolar	Ethylene production and signaling in plants	[197]

## Abbreviations

NOD: Nitric-oxide dioxygenase
Hb: Hemoglobin
Mb: Myoglobin
flavoHb: Flavohemoglobin
SDG: Single domain globin
Cygb: Cytoglobin
Ngb: Neuroglobin
trHb: Truncated hemoglobin
nsHb: Non-symbiotic hemoglobin
SOD: Superoxide dismutase
NOR: Nitric-oxide reductase
NOS: Nitric-oxide synthase
sGC: Soluble guanylate cyclase
IRE-BP: Iron responsive element binding protein.
